# Analysing multiple types of molecular profiles simultaneously: connecting the needles in the haystack

**DOI:** 10.1186/s12859-016-0926-8

**Published:** 2016-02-09

**Authors:** Renée X. Menezes, Leila Mohammadi, Jelle J. Goeman, Judith M. Boer

**Affiliations:** Department of Epidemiology and Biostatistics, VU University Medical Center, De Boelelaan 1089a, HV Amsterdam, 1081 The Netherlands; Department of Statistics, Shiraz University, Shiraz, Iran; Biostatistics, Department for Health Evidence, Radboud University Medical Center, Nijmegen, The Netherlands; Medical Statistics and Bioinformatics, Leiden University Medical Center, Nijmegen, The Netherlands; Department of Pediatric Oncology and Hematology, Erasmus MC-Sophia Children’s Hospital, Rotterdam, The Netherlands; Netherlands Bioinformatics Centre, Nijmegen, The Netherlands

**Keywords:** Gene set, Integration, “ *p*>>*n*”, Global test, Penalized regression

## Abstract

**Background:**

It has been shown that a random-effects framework can be used to test the association between a gene’s expression level and the number of DNA copies of a set of genes. This gene-set modelling framework was later applied to find associations between mRNA expression and microRNA expression, by defining the gene sets using target prediction information.

**Methods and results:**

Here, we extend the model introduced by Menezes et al. 2009 to consider the effect of not just copy number, but also of other molecular profiles such as methylation changes and loss-of-heterozigosity (LOH), on gene expression levels. We will consider again sets of measurements, to improve robustness of results and increase the power to find associations. Our approach can be used genome-wide to find associations and yields a test to help separate true associations from noise.

We apply our method to colon and to breast cancer samples, for which genome-wide copy number, methylation and gene expression profiles are available. Our findings include interesting gene expression-regulating mechanisms, which may involve only one of copy number or methylation, or both for the same samples. We even are able to find effects due to different molecular mechanisms in different samples.

**Conclusions:**

Our method can equally well be applied to cases where other types of molecular (high-dimensional) data are collected, such as LOH, SNP genotype and microRNA expression data. Computationally efficient, it represents a flexible and powerful tool to study associations between high-dimensional datasets. The method is freely available via the SIM BioConductor package.

**Electronic supplementary material:**

The online version of this article (doi:10.1186/s12859-016-0926-8) contains supplementary material, which is available to authorized users.

## Background

The wealth of omics data being currently produced raises the need for efficient and flexible models to analyse these data. One typical objective is to understand which molecular changes affect gene expression levels or, if available, protein expression levels. Molecular profiles reliably measured currently include DNA methylation and copy number, sequence information including SNP and loss-of-heterozigosity (LOH) information, as well as microRNA expression levels. All these are known to be involved in gene expression regulation.

Many methods have so far been proposed for finding associations between two omics data sets (for an overview see [1]). These enable us to study for example which copy number changes affect gene expression levels, or alternatively which methylation changes affect gene expression, and to which extent they do so. Such findings can for example shed light onto oncogenic mechanisms. One such method has been proposed by Menezes et al. [2], whereby a statistical test is used to for example assess the association between gene expression and the copy number of a genomic region around it. In that work, the authors showed the power gain to find associations yielded by considering sets of measurements, rather than considering only associations between pairs of features, as done elsewhere [3].

Methods have also been proposed for the joint analysis of microRNA expression and mRNA expression profiles. In this context, many microRNAs can target the same mRNA, and one microRNA can target multiple mRNAs. So it makes sense to consider target prediction information while looking for associations between mRNA and microRNA expression levels. van Iterson et al. [4] use the method proposed by Menezes et al. [2], with gene sets now defined by various target prediction tools. It is then shown that the method has power to find associations that are sucessfully validated, in spite of limited sample size. They also show that predicted associations using sets of features lead to more robust, and thus more reliable, results, in this case with a higher validation rate, than associations predicted using pairs of features (i.e. one miRNA and one mRNA at a time). This is in agreement of findings from Menezes et al. [2]. The extra robustness is brought in by the fact that the impact on small effects observed for specific genes must be observed for at least a considerable subset of genes, before they become significant.

With multiple types of molecular profiles measured, it makes sense to consider methods for analysing all of them together. Only a few of the integrated analysis methods so far proposed have been extended to handling more than two data sets. Methods proposed by Waaijenborg et al. [5] and by Witten et al. [6] use a sparse canonical correlation framework. As such, they are of an exploratory nature, aiming at finding sets of covariates from the various data sets which are most correlated.

Here we extend the integrated analysis method proposed by Menezes et al. [2] to handle multiple high-dimensional data sets. The aim is to test for association between one type of molecular profile (mRNA, say) and other types (copy number and methylation, say), the latter represented by sets of probes, rather than individual ones. Under the null hypothesis, no association exists between mRNA and either copy number or methylation, in our example. The use of sets makes for a robust and powerful method: robust, because noise originating from individual probes is ignored; and powerful, because subtle associations found between mRNA and multiple methylation probes are detectable as the probability of seeing many of these small associations together by chance will be considered small, which would be ignored if they were considered separately. Given the high-dimensionality of the problem, the fact that our method evaluates statistical significance is crucial to help separate noise from true associations. Moreover, since our method uses a regression framework, it can take confounders into account. As the original method, it is thus a flexible, powerful and efficient method to analyse jointly many omics data sets.

This paper is organized as follows. In the “Methods” section we present the statistical test for associations between a response and multiple gene sets. Here we will refer to *covariate sets* instead of *gene sets*, essentially because sets may contain any set of variables, and need not refer to a gene. In the “Results” section we illustrate the workings of our method under various types of associations between data sets with a simulation study. We also apply our method to sets of TCGA samples: 125 of colon cancer and 173 of breast cancer, for which copy number, methylation and gene expression profiles are available.

## Methods

### The integrated analysis model

Menezes et al. [2] have proposed using score tests to find associations between a response (say, the expression levels of a gene) and a set of covariates (say, the genomic copy number measured at multiple loci on the same chromosome arm as the gene). Let us represent by *Y*_*ni*_ the expression for gene *i*, and by *X*_*nj*_ the genomic copy number for gene *j*, where $i=1,\dots,I$ and $j=1,\dots,J$ represent the sets of probes used, and $n=1,\dots,N$ indexes the sample. Note that it is assumed that measurement sets {*Y*_*i*_},{*X*_*j*_} are available per sample, although it is not necessary that both sets of probes correspond to the same loci. Then we write, for any given *i*,

(1) $$ E\left(Y_{ni}\right) = h\left(\alpha + \sum_{j=1}^{J} \beta_{j} X_{nj} \right),\ n=1,\dots,N,  $$

where *h*(.) is a given inverse link function and $\{\beta _{j}\}\sim {\mathcal {N}}(0,\tau)$, making model (1) a random-effects model. From now on, we consider observations *Y*_*i*_ for a single gene indexed by *i*, but we omit the index *i* for clarity.

The approach proposed by Menezes et al. [2] focuses not on fitting model (1) directly, but on testing whether or not the association between *Y* and {*X*_*j*_} is statistically significant, for each gene expression probe *Y*. This is done by making use of the global test [7]. In this framework, under the null hypothesis that *Y* is not associated with the set {*X*_*j*_}, we have Var(*β*)=*τ*=0. On the other hand, when *Y* displays association with variables in {*X*_*j*_}, then some of the *β*_*j*_ will be non-zero, and thus Var(*β*)>0. Specifically, the global test is a score test for the hypotheses

$$H_{0}: \text{Var}(\beta) = 0 \quad \mathrm{vs.}\quad H_{a}: \text{Var}(\beta) \neq 0, $$

first proposed by [8] and later applied to the context of testing association between a molecular profile and a clinical variable by Goeman et al. [7]. Let us define $r_{n}=\sum _{j} \beta _{j} X_{\textit {nj}}$, the part of the linear predictor that depends on the data, $r=(r_{1},r_{2},\dots,r_{N})^{t}$, and *X* an *N*×*J* matrix containing all observations for the covariates. Then a statistic to test the hypotheses above was proposed by Goeman et al. [7] as

(2) $$ Q(X) \propto \frac{(Y-\mu)^{t} X X^{t} (Y-\mu)}{(Y-\mu)^{t} (Y-\mu)},  $$

or in its standardized form

(3) $$ T = \frac{Q(X)- E\left[Q(X)\right]}{\sqrt{\text{Var}\left[Q(X)\right]}}.  $$

Applying this test generates one *p*-value per gene expression variable *Y*. For a set of gene expression probes $\{Y_{i},\ i=1,\dots,I\}$ a list of *Ip*-values is obtained.

### Extension to two sets of covariates

Assume now that a second set of covariates {*Z*_*k*_} is observed, and there is interest in studying the association between both covariate sets and the response *Y*_*i*_. Now model (1) becomes

(4) $$ E\left(Y_{ni}\right) = \alpha + \sum_{j=1}^{J} \beta_{j} X_{nj}+ \sum_{k=1}^{K} \gamma_{k} Z_{nk},\ n=1,\dots,N,  $$

where $\{\beta _{j}\}\sim {\mathcal {N}}(0,\sigma)$ and $\{\gamma _{k}\}\sim {\mathcal {N}}(0,\tau)$, so that model (4) still is a random-effects model, and where we have assumed for simplicity that *h*(*x*)=*x*. Similarly to the single covariate-set case, under the null hypothesis no association exists between either *Y* and {*X*_*j*_}, or *Y* and {*Z*_*k*_}. In this case, obviously the variances *σ*,*τ* of the random effects {*β*_*j*_},{*γ*_*k*_} in (4) must be zero. On the other hand, under the alternative hypothesis, *Y* displays association with either {*X*_*j*_} or {*Z*_*k*_}, meaning that either one of the two random-effect variances *σ*,*τ* must be non-zero. Thus, to test for association between each *Y*_*i*_ and the sets of variables {*X*_*j*_},{*Z*_*k*_}, it is of interest to test the hypotheses

$$\begin{array}{@{}rcl@{}} H_{0}:&& \text{Var}(\beta) = \text{Var}(\gamma) = 0 \quad \mathrm{vs.}\quad \\ H_{a}:&& \text{Var}(\beta) \neq 0\ \text{or}\ \text{Var}(\gamma) \neq 0 \end{array} $$

To derive the test statistic in this case, we first write the likelihood for {*Y*_*i*_} as

(5) $$ L(\boldsymbol{\beta},\boldsymbol{\gamma},\sigma,\tau) = \mathrm{E}_{r}\left[ \prod_{i=1}^{I} f_{i}\left(Y_{i} | r_{i},\boldsymbol{\beta},\boldsymbol{\gamma},\sigma,\tau \right) \right],  $$

with *f*_*i*_ representing the density of *Y*_*i*_, given the random effect *r*_*i*_=*X*_*i*_***β***+*Z*_*i*_***γ***, and it is assumed that Cov(***β***)=*σI*, Cov(***γ***)=*τI* and Cov(***β***,***γ***)=0. Using a Taylor series, we can derive a first-order approximation for the term in the expectancy as:

(6) $$\begin{array}{@{}rcl@{}} && \left[ \prod_{i=1}^{I} f_{i}(0) \right] \left\{ 1 + \frac{1}{2}\sum_{i=1}^{I}\left(\sigma R_{ii} + \tau S_{ii} \right) {u_{i}^{2}}(0)\right. \\ && \left.+ \frac{1}{2}\sum_{i=1}^{I}\sum_{j=1}^{I} \left(\sigma R_{ij} + \tau S_{ij} \right) {u_{i}^{1}}(0) {u_{j}^{1}}(0) \right\}. \end{array} $$

Let us define ***θ***=(*θ*_1_,*θ*_2_)≡(*σ*,*τ*). By assuming that *Y*_*i*_ has a distribution belonging to the exponential family with canonical link, we obtain expressions for partial derivatives of the log-likelihood with respect to *σ*,*τ*. With some algebra, we get that the score vector **U**(***θ***) is proportional to

(7) $$ \left[ \left\{ Q(R) - \text{tr}(RV) \right\}, \left\{ Q(S) - \text{tr}(SV) \right\} \right],   $$

where *R*≡*XX*^*t*^ and *S*≡*ZZ*^*t*^. From [7] we know that *E*[*Q*(*R*)]=tr(*RV*) and, similarly, *E*[*Q*(*S*)]=tr(*SV*). A detailed derivation is given in Section 2 of the Additional file 1. Thus, the score vector is formed by the centered and unscaled individual test statistics, for association between *Y* and *X*,*Z* separately. It then follows that a test statistic to test the hypothesis *H*_0_: *σ*=*τ*=0 can be obtained by using **U**(***θ***_0_)^*t*^**U**(***θ***_0_), leading to the expression

(8) $$ \left[ Q(R) - \text{tr}(RV) \right]^{2} + \left[ Q(Z) - \text{tr}(SV) \right]^{2}.  $$

Note that this is not the score test statistic, although it is derived from the score vector. This is similar to the suggestion in Goeman et al. [7] of using only centering while testing for association with a single set *X*. For simplicity, we may ignore the centering and the squaring, then write the unscaled test statistic for two covariate sets as

(9) $$ Q(X,Z) \equiv Q(X) + Q(Z).  $$

It is interesting to note that, from (2),

$$Q(X) + Q(Z) = \frac{(Y-\mu)^{t} \left[ X X^{t} + Z Z^{t} \right] (Y-\mu)}{(Y-\mu)^{t} (Y-\mu)}, $$

where *XX*^*t*^+*ZZ*^*t*^ is the matrix in the quadratic form that would have been obtained if our model had a single set of covariates given by the merged set {*X*,*Z*}, and with effect modelled by a single vector of random effects. In such a case, the design matrix would have been obtained by binding the columns of *X* and *Z* together and, thus, the unscaled score test statistic would be given as above.

The same test statistic (9) could have been derived by taking *σ*=*η*∗*τ*, where *η* is a given constant, and deriving the test in terms of *τ* only. The choice of *η* is free but must be made *a priori*, each choice resulting in a test with different power properties. It is intuitive to see that this is possible, and that the derivation used here corresponds to choosing *η*=1, by the equivalence to a test for association with a single set of covariates.

Thus, a test statistic for two covariate sets can be obtained as the sum of the individual (centered) test statistics per covariate set, as in (8). Similarly for the unscaled and unsquared versions of the score test statistics, the test statistic for two covariate sets *X* and *Z* is equivalent to the one obtained for a single covariate set formed by {*X*,*Z*}.

In practice, some sort of centering and scaling of *Q*(*X*),*Q*(*Z*) may be used, especially when covariates take values in different ranges, are of very different sizes and/or display different variances. In such cases, centering and scaling can ensure the separate sets are given the correct weights in the combined test statistic *Q*(*X*,*Z*). This can be done by using the scaled individual test statistics as in (8). An alternative is given in Section 5 of Additional file 1.

### Extension to more than two covariate sets

Since the inner product of the score vector is equal to the sum of the squared and centered test statistics for the individual covariate sets, for any given number of covariate sets *M*, the test statistic (8) obtained for *M*=2 can be extended for any *M*. In particular it is straightforward to show that, if squaring and centering are ignored, the test statistic for any *M* number of covariate sets is the same one as generated by a model with a single covariate set, formed by merging all covariate sets together.

### Data correlation structure

We have seen that the unscaled and linear form of the test statistic (9) equals the test statistic for a single covariate set, formed by the union of all covariate sets into a single one, or *Q*(*W*) where *W* is a matrix formed by the columns of *X*,*Z* bound together. As such, the test statistic proposed considers not only correlation between the covariates and the dependent variable *Y*, but also pairwise correlations between covariate pairs, as shown by Goeman et al. [9], section 7. In particular, this means that the test statistic will have most power in directions where {*X*,*Z*} have the most (co)variance, as well as display association with *Y*. The specific directions where most power lies will depend on the correlation structure in the data. In the “Results” section, we will illustrate in a simulation study how the power varies, considering correlation structures of practical interest.

### Test statistic null distribution

For testing, the distribution of $T_{\textit {XZ}}^{2}$ under the null hypothesis of no association between *Y* and *X*,*Z* is needed. Here we will consider the expression (9) for the combined test statistic. For the single-set testing, Goeman et al. [10] obtains an expression for the asymptotic distribution of *Q*(*X*) under a generalized linear model, which is the exact finite sample distribution under the linear model, as used here. This (asymptotic) null distribution can be written as a ratio of weighted sums of ${\chi ^{2}_{1}}$ random variables [10].

Our test statistic (9) for two covariate sets *X*,*Z* can be seen as a test statistic for a single covariate set resulting of the union of the two original sets. This means that the distributions derived in Goeman et al. [10] can be used for (9).

Note that, in case of applying the test to many separate responses {*Y*_*i*_}, such as expression levels of many different genes, the resulting computational burden of numerically estimating the distribution per response may be superior to computing *p*-values via permutation. In the cancer examples, we use permutations to compute *p*-values for *Q*(*X*,*Z*) for computational ease, and use the sum of test statistics (9).

### Software

Methods presented in this work are implemented in the Bioconductor package SIM, currently for a single covariate set, and in the short-term for multiple covariate sets. All computations described and applied in this work used R from at least version 3.0.1 (see [11] for a recent reference).

## Results

### Simulation study

#### Setup

We run a simulation study to evaluate the power of the proposed test statistic under various types of effects. A detailed description of the entire study setup is given in Section 1 of the Additional file 2. For completeness, here follows a brief description. The data here is assumed to consist of two explanatory sets of covariates, $\{X_{j},\ j=1,\dots,J\}$ and $\{Z_{k},\ k=1,\dots,K\}$, and a set of dependent variables $\{Y_{i},\ i=1,\dots,I\}$.

We consider four independent data sets, each involving one set of variables {*Y*_*i*_,*X*_*j*_,*Z*_*k*_}, with $i,j,k=1,\dots,1000$. Each data set can be seen as a (genomic) region, here assumed to involve one association type between the covariate sets and the dependent variable *Y*_*i*_ for $i=1,\dots,500$, and no association for the remaining probes. The associations considered are: region I, where *Y*_*i*_ is associated with {*X*_*j*_} only, which we will refer to as “x only”; region II, “additive”, where both covariate sets affect outcome linearly; region III, “multiplicative”, where both covariate sets affect outcome linearly as well as multiplicatively; and region IV, “split-samples” or “complementary”, where {*Y*_*i*_} depends upon {*X*_*j*_} for half of the samples, and for the other half {*Y*_*i*_} depends upon {*Z*_*k*_}. For each data set, three sample sizes are considered: 50, 100 and 200 samples.

Within each region, we test for association between each *Y*_*i*_ and the covariate sets {*X*_*j*_},{*Z*_*k*_} using the test statistic (8). Here *p*-values are estimated by comparing the observed test statistic to values obtained after permuting the dependent variable samples 1000 times, and re-computing the test statistics. For the aims of this particular study, which is to compare ROC curves, no multiple testing is necessary.

In what follows, we introduce correlation between *X* and *Z* in two different ways. Firstly we define each entry in *Z* as a linear function of the corresponding entry in *X*, with remaining entries uncorrelated. Subsequently, we consider a more realistic framework by assigning empirical correlation structures to *X*,*Z*. More details about the entire study setup are given in Section 1 of the Additional file 2.

#### *Z* is a function of *X*

Here we assume that $X_{j}\sim {\mathcal {N}}(1,2.25)$ for all *j*, with all *X*_*j*_ independent, and *Z*_*k*_≡*X*_*k*_+*W*_*k*_, with $W_{k}\sim {\mathcal {N}} (0,\gamma)$, and *W*_*k*_⊥*X*_*k*_, for all *k*. For simplicity we assume that *I*=*J*=*K*. The relationship between *X*_*j*_ and *Z*_*k*_ implies that Var(*Z*_*k*_)=Var(*X*_*k*_)+Var(*W*_*k*_) and Cor(*X*_*k*_,*Z*_*k*_)={Var(*X*_*k*_)/[Var(*W*_*k*_)+Var(*X*_*k*_)]}^1/2^. The amount of correlation between *X*_*k*_ and *Z*_*k*_ can be changed by simply changing Var(*W*_*k*_. For the particular parameter values chosen here, we would get Cor(*X*_*k*_,*Z*_*k*_)=0.83 if Var(*W*_*k*_)=1 and Cor(*X*_*k*_,*Z*_*k*_)=0.56 if Var(*W*_*k*_)=5, for all *k*, whilst Cor(*X*_*j*_,*Z*_*k*_)=0 for *j*≠*k*, although observed values may vary due to randomness.

We will also simulate data with the same assumptions as above, but now take *Z*_*k*_≡−*X*_*k*_+*W*_*k*_, so that *X*_*k*_,*Z*_*k*_ have a negative correlation. For this case, we will take Var(*W*_*k*_)=1, so that Cor(*X*_*k*_,*Z*_*k*_)=−0.83. Note that the {*X*_*j*_} are uncorrelated, as are {*Z*_*k*_}, in all three cases.

**Fig. 1:**
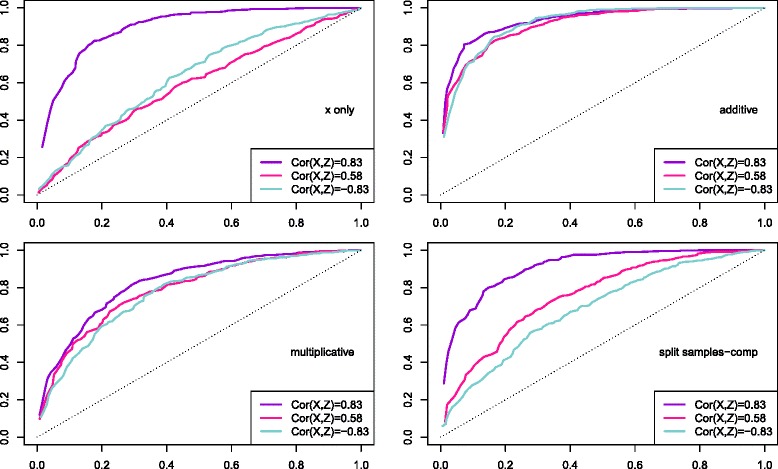
ROC curves, simulation study with *Z* as a function of *X*. ROC curves to evaluate power to find four different types of effects between one dependent variable and two independent covariates sets, *N*=100. Simulation Study using *Z* as a function of *X*. Different line colours correspond to different amounts of correlation between *Z* and *X*

ROC curves produced for each different type of effect/region suggest that most power is typically yielded when the covariate sets *X* and *Z* are positive and strongly correlated (Fig. 1 for *N*=100, Additional file 2: Figures S1 and S2 for *N*=50,200). The biggest difference in power is observed in the situation where *X* affects the response, followed by the split-samples case. This is surprising, but not entirely unexpected. Indeed, in this case *Q*(*Z*) brings information about *Q*(*X*) and thus, when their sum is used for testing, the power to find effects increases the most.

#### Using empirical correlation structures

In practice, correlation structures can be considerably more complex than assumed in the previous subsection, so we here consider two empirical correlation matrices, derived from the TCGA colon cancer data studied in the subsection “Colon and breast cancer datasets”. In this example, gene expression is explained by set of copy number (CN) or methylation (ME) covariates. Specifically, we chose genes *GGT7* and *GDAP1L1*, both on 20q. Expression of both genes was found to be associated with CN as well as ME in our analyses (see “Examples of effects found” later on). For *GGT7*, tests involved 21 CN and 6 ME covariates. The majority of copy number and methylation covariates were negatively associated with each other, with the strongest Pearson correlation estimated as −0.83. Gene *GDAP1L1* was found to have expression associated with 20 CN and 2 ME covariates, but here CN and ME covariates are positively associated with each other, with Pearson correlation around 0.5. Subsequently, observations for {*X*_*j*_} and {*Z*_*k*_} are generated from a multivariate normal distribution based upon one of the covariance matrices at a time. For both genes, CN measurements are highly associated with each other (Pearson correlations ≥ 0.9 in general).

**Fig. 2:**
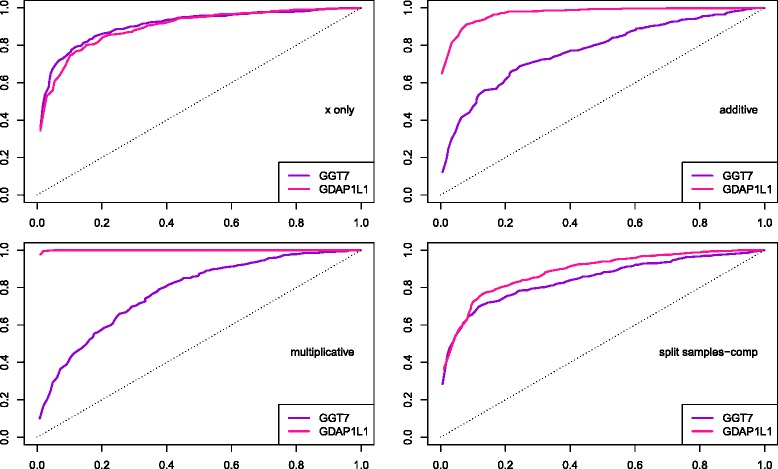
ROC curves, simulation study with empirical correlation matrix for genes *GGT7* and *GDAP1L1*. ROC curves to evaluate power to find four different types of effects between one dependent variable and two independent covariates sets, *N*=100. Simulation Study using an empirical correlation matrix for *X*,*Z*. Different line colours correspond to different correlation matrices, corresponding to the covariate sets for the genes considered

The ROC curves suggest that power achieved is comparable in the two cases, for situations where only one of the covariate sets is correlated with the response, or where the covariate sets are correlated to different subsets of observations for the response (Fig. 2, *N*=100). However, when effects are either additive or multiplicative, then power to find effects is greater if the covariate sets are positively correlated, as is the case with *GDAP1L1*. Conclusions remain the same for *N*=50,200 (data not shown).

We conclude that the test statistic (8) yields acceptable power to find associations with the response, in situations of practical interest.

### Colon and breast cancer datasets

#### Data and definitions

In our simulation studies we have shown that the joint test statistic *Q*(*X*,*Z*) given by (8) can test for, and find, different types of associations between covariate sets and responses. Here we will see that its relationship with the individual test statistics *Q*(*X*),*Q*(*Z*) can help elucidate the relative effects of molecular mechanisms under study on the response. To illustrate this, we will consider molecular profiling (mRNA, DNA copy number and methylation) data for colon (*N*=125) and breast (*N*=173) cancer samples, extracted from The Cancer Genome Atlas (TCGA). For details about the data, see Section 2.1 of Additional file 2.

Here we will consider gene expression as the response variable, and we will study how DNA copy number and methylation affect expression levels, both individually as well as jointly. Specifically, we consider a model such as (4), where *Y*_*i*_ represents the *i*th gene expression probe, {*X*_*j*_} represents the set of copy number probes that are within 1 Mb in either direction of the transcription start site of the gene, and {*Z*_*k*_} represents the set of methylation probes that are within 50 Kb of the gene’s transciption start site in either direction. These window sizes are arbitrary and reflect current knowledge on the distance of *cis*–regulatory effects of copy number ([3, 12]) and methylation on gene expression [13].

We computed *p*-values for *Q*(*X*,*Z*), which we will refer to as the joint test, as well as for the individual test statistics *Q*(*X*),*Q*(*Z*), which will be referred to as the CN test and the ME test, for association between DNA copy number or methylation with the gene expression. In all comparisons, we use *p*-values not corrected for multiple testing, as the comparisons between different test results can be more reliably done in this way (in practice, multiple testing should always be used). Associations for which *p*≤0.001 were selected. In all cases, empirical *p*-values distributions were verified to be enriched with small *p*-values, so that in none of the cases does the set of mRNA probes selected have small *p*-values entirely due to chance. Indeed, if we consider the number of tests done per chromosome arm (Additional file 2: Table S3), we can verify that the largest number of *p*-values that by chance is smaller than 0.001 is 4, for 1p. Various ratios of the number of selected tests will be computed, following definitions in Additional file 2: Table S4

Tables of all mRNA probes tested, with *p*-values computed by joint and individual tests, can be found in Additional file 3 for the colon cancer data, and Additional file 4 for the breast cancer data.

#### Individual and joint copy number and methylation effects

Individually, copy number changes explained a larger portion of the gene expression variance than methylation, notably for the colon cancer data set (see Additional file 2: Table S5). Interestingly, in the colon cancer data we found twice as many mRNA expression probes selected as associated with copy number (16 % of the total) than in the breast cancer data (8 %), despite the latter involving almost 50 % more samples than the former (173 and 125, respectively). So, copy number changes seemed to regulate the expression of a larger number of genes for colon cancer, compared with breast cancer. The proportion of mRNA expression probes selected as being regulated by methylation changes was comparable in these two data sets (5.4 and 5.2 % for colon and breast, respectively).

**Fig. 3:**
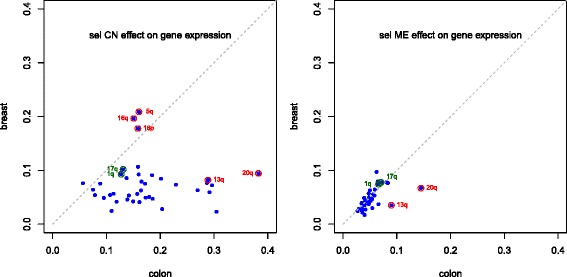
Comparison of copy number and methylation effects. Proportion of genes selected by their expression association with copy number (*left*) and with methylation (*right*), for colon (x-axis) and breast (y-axis) cancers, per chromosome arm

Results separately per chromosome arm mostly reflect the stronger copy number effect in colon cancer compared with breast cancer. Methylation effects are, on the other hand, observed in very similar proportions (Fig. 3 and Additional file 2: Table S5 and S6). Examples of this are found with 1q and 17q. However, two chromosome arms display more extreme behaviour: 20q and, to a lesser extent 13q, are outliers in the colon cancer data set, with a larger proportion of selected associations with either copy number or methylation than the remaining arms.

Proportions of selected joint tests for colon and breast cancer (Additional file 2: Figure S3) reflect mostly the proportions found with the CN test (Fig. 3, Left graph); this is due to the copy number effect largely influencing the joint effect.

We can better understand these differences if we now consider test results per gene. For colon cancer, the joint test yielded virtually the same results as the CN test, for 20q and 13q in colon cancer (see top-left hand-side graphs of Additional file 2: Figure S4 and S5). In addition, more than 76 % of all mRNA probes selected by ME tests were also selected by CN tests (Additional file 2: Figure S6). As both 13q and 20q often display copy gains in colon cancer (which is the case in this data), this suggests that those gains are likely to be important for oncogenesis, and methylation may be compensating for some of this effect. In contrast, for breast cancer the joint test selects mRNA probes that were not selected by the CN test on 20q (Additional file 2: Figure S4, top-right), and at most 37 % of the mRNA probes selected by ME tests were also selected by CN tests (Additional file 2: Figure S6 and Table S8).

On the other hand, genes on 5q, 16p and 16q displayed associations mostly with copy number, but not with methylation, for both cancer types (Fig. 3). Here, the joint test selects additional mRNA probes, not selected by the individual test statistics (see Additional file 2: Figure S7 for 5q – results for 16p and 16q are similar and not shown). In addition, mRNA probes selected by ME tests were selected also by the CN test less often (18–35 % in colon cancer, 52–64 % in breast cancer – Additional file 2: Figure S6). This makes results for 13q and 20q in colon cancer more remarkable: they yielded the highest percentages of selected CN tests and ME tests, and the largest overlap of ME tests with CN tests.

Such comparisons help picking up trends that differentiate cancer types, although they obviously ignore region-specific effects: 17q has commonly amplified regions in breast cancer with an impact on gene expression [2], which is not the case for colon cancer.

#### Overlap between individual and joint tests

Results from joint and individual tests may naturally display some association: this indicates how much of the total gene expression variability is explained by each individual effect. Here we considered the proportion of tests selected both individually as well as with the joint test, compared to the total of selected joint tests (“CN and joint overlap” and “ME and joint overlap”, in Additional file 2: Table S4–S7). When the joint test leads to many extra discoveries compared with the individual test, this proportion is small. If the proportion is close to 1, however, the joint test statistic mostly finds associations already identified by the individual test, suggesting that a single molecular profile drives effects on gene expression.

**Fig. 4:**
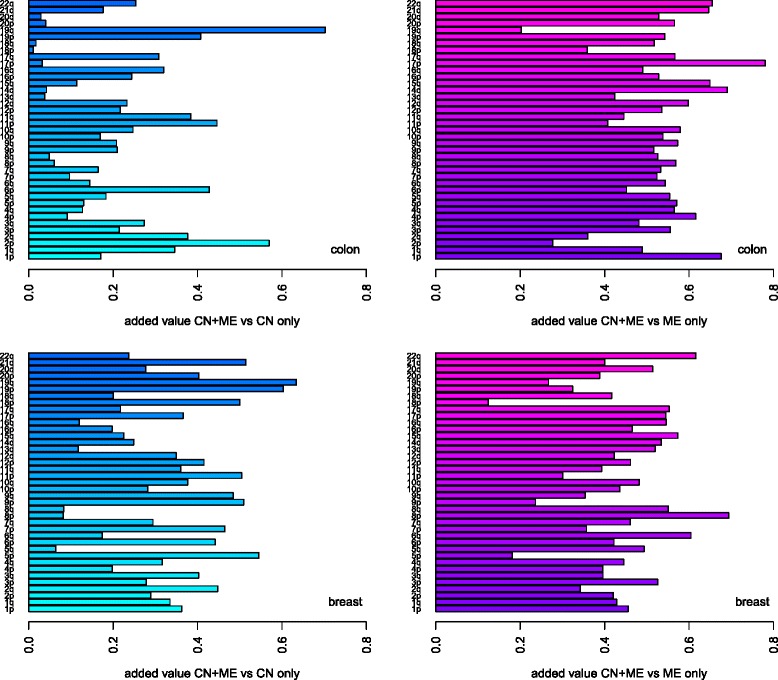
Joint test new discoveries. Computed as the proportion of genes with selected expression association with the joint set of covariates, that was found not to be selected by either copy number (left-hand side, blue shades) or methylation (right-hand side, purple shades) separately. *Top*: colon cancer. *Bottom*: breast cancer

The complement of this proportion (obtained by computing 1-proportion), the ratio of new discoveries with the joint test, points out clearly that large fractions of joint test new discoveries arise, when compared with the ME test (Fig. 4). This was expected: most of these result from the stronger copy number effect. Nevertheless, the advantage of the joint test is evident as there are new discoveries with the joint test for all chromosome arms.

The overlap ratios show that, as expected, a relatively large proportion of mRNA probes selected by joint tests was also selected by the CN test (80 and 70 % for colon and breast, respectively — Additional file 2: Table S5). In contrast, selected joint tests were also selected with the ME test at considerably smaller proportions (29 and 41 % for colon and breast, respectively — Additional file 2: Table S5). This can be again explained by the stronger copy number effects in colon cancer, and stronger methylation effects in breast cancer. Note that herewith when we refer to a “selected test” we obviously mean a “selected mRNA probe by the test”.

**Fig. 5:**
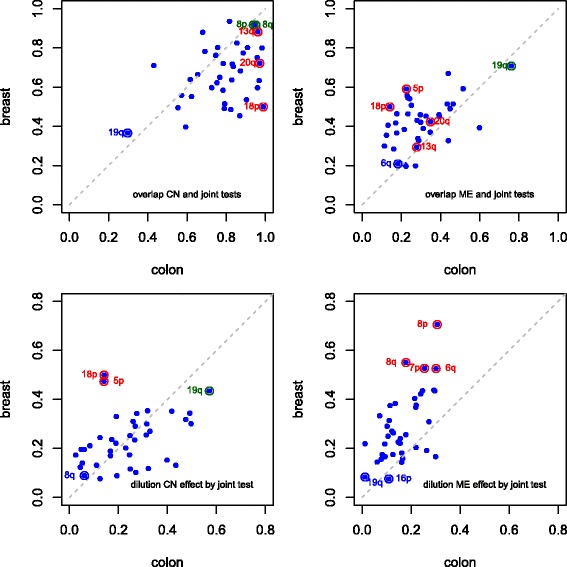
Comparing individual and joint tests: overlap and dilution. *Top* row: overlap between individual and joint tests. Computed as the proportion of genes selected by both its expression being associated with the joint set of covariates, formed by both copy number and expression, as well as by each single set of covariates, per chromosome arm. This proportion is relative to all selected joint tests. *Bottom row*: dilution. Computed as the proportion of selected genes for expression association with one set of covariates (either copy number or methylation), that are not selected with the joint set of covariates, formed by both copy number and expression, per chromosome arm. The proportion is relative to all selected tests for association with either copy number (*bottom-left*) or methylation (*bottom-right*)

Results per chromosome arm mostly reflected the strong copy number effect, especially for colon cancer (Fig. 5, graphs on top row). Some special patterns appear: 8p and 8q display similar effects in both cancers as 13q and 20q in colon: selected joint tests were mostly also selected by the CN test (Additional file 2: Table S6 and S7), and most selected ME tests corresponded to selected CN tests (see Additional file 2: Figure S6 for an overview, and Additional file 2: Figure S8 for 8q).

Another interesting case is 18p, where copy number changes seemed to drive mRNA expression regulation, with virtually all (99 %) selected joint tests overlapping with selected CN tests for colon cancer. For breast cancer, selected joint tests corresponded to selected CN and ME tests in precisely 50 % of the cases each, with no overlap between selected CN and ME tests (Additional file 2: Figure S6). Thus, here copy number and methylation changes seem to drive gene expression regulation, independently of each other, for different genes.

Another interesting example is 19q, for which approximately 63–70 % of the selected joint tests were not selected by the CN test individually, whilst selected joint and ME tests had the largest overlap (76–70 %), suggesting that methylation effects may drive gene expression regulation on 19q, for both cancers. Indeed, methylation effects dominated the joint test statistic value, although copy number effects were still found for genes not found to be associated with methylation change (Additional file 2: Figure S6 and S9).

Methylation seemed to play a stronger role in gene expression regulation in breast cancer, compared with colon cancer. This was particularly noticeable examining the overlap proportions between selected joint and methylation tests. On chromosome 9, in particular, these proportions of overlap were strikingly larger in the breast cancer data (55 and 51 % for 9p and 9q, respectively), compared with the colon cancer data (23 and 21 % for 9p and 9q – see Additional file 2: Table S6 and S7). Interestingly, one of the selected genes on 9p is *CDKN2A*, which is mapped by six mRNA probes on the Agilent expression microarray used. Of these, two were selected as associated with both the methylation and the joint tests in the breast cancer data, and two were just above the selection threshold (*p*-values ≤ 0.005) with these two tests. In contrast, in the colon cancer data only one probe would be selected with a less stringent cut-off, by the methylation and the joint tests (*p* ≤ 0.005). Indeed, methylation and gene expression were associated in both data sets, although the correlation was more widely and evenly spread across both methylation as mRNA-expression probes (Additional file 2: Figure S10).

In order to better understand the mechanisms regulating *CDKN2A* expression, let us consider the mRNA probe A_23_P17356 which had significant associations in breast, but not colon, cancer. A total of 49 breast cancer samples displayed DNA copy loss, whilst only 6 colon cancer samples displayed a small loss in this region (Additional file 2: Figure S11–S12, top). Note that the mRNA probe was often not under-expressed for these samples. After sorting samples in the methylation data according to the copy number data heatmaps, we see that the samples displaying larger DNA copy loss (right-hand side) also displayed hypo-methylation of about half of the probes which, again, was only observed for breast cancer (Additional file 2: Figure S11–S12, bottom). We then looked at scatterplots of A_23_P17356 expression and the 19 methylation probes in the covariate set tests, which suggested that many of those displayed negative association with mRNA expression in breast cancer, but not in colon, as expected of a functional hyper-methylation event affecting gene expression (Additional file 2: Figure S13–S14). In particular, DNA loss was associated with hypo-methylation of some probes in breast cancer, but these probes were not correlated with mRNA expression. Indeed, methylation probes correlated with mRNA expression did not show correlation with DNA copy number. This suggests that, in breast cancer, methylation affects *CDKN2A* expression, independently of DNA copy number change.

#### Tests with different conclusions

A selected joint test typically corresponded to at least one selected individual test, making interpretation of results straightforward. However, there was a small but non-negligible proportion of mRNA probes (0.6 % for colon and 1.2 % for breast) that was selected by the joint test, whilst that was not the case with either of the individual tests (see line “Joint sel but not CN ME/joint sel” in Additional file 2: Table S4–S5). Such cases tended to reflect small effects spread across multiple covariates, in this case both copy number and methylation probes, with the collection of small effects leading to an association with the larger covariate set being selected. As such, these are worthy of further investigation. Looking at the results per chromosome arm, we found that 11p and 17p had relatively high proportions (4 and 3 % respectively) of effects found only with the joint test statistic, for the breast cancer data (see Additional file 2: Figure S15 and Table S7).

There were also mRNA probes selected only by an individual test, but not by the joint test. Indeed, around 21 % of the mRNA probes selected by CN tests were not selected by the joint test, for both colon and breast cancers. If such effect dilution occurs, the conclusion is obviously that copy number affects gene expression whilst methylation does not, to an extent that the copy number effect, either not very strong or only spanning a small subset of the copy number variables, is no longer enough to drive the result of the joint test statistic. From the mRNA probes selected by ME tests, between 15 and 27 % were found not to be selected by the joint test, for colon and breast cancer respectively.

Copy number effects were found to be up to 40 % diluted for most arms (Fig. 5, bottom-left). This was partly expected due to the large overlap between joint and CN tests. On exception is 19q, of all mRNA probes selected by the CN test, around 57 % for colon and 43 % for breast were not selected by the joint test statistic, whilst for the ME test these proportions were 1 and 8 % respectively (Fig. 5, bottom-right). So, 19q is a chromosome arm where methylation effects dominated the joint test statistic and, as such, were diluted to a very small extent; on the other hand, about half of the individual copy number effects were diluted in the joint test statistic.

Overall, we observed more dilution of methylation effects in the joint test statistic with the breast cancer data, compared with colon (bottom-right hand-side graph in Fig. 5). This is likely due to the fact that the breast cancer displayed more methylation effects but, as these effects were mild, they were more likely to be diluted in the joint test statistic. The largest methylation-effect dilutions in the breast cancer data were observed for 6q, 7p, 8p and 8q, where the last two yielded at least 92 % of selected joint tests also being selected by the CN test, for both colon and breast cancer. What the dilution proportions tell us is that the copy number effect dominance comes at the cost of the methylation effect: between 18 and 31 % in colon cancer, and between 53 and 71 % in breast cancer, of the methylation effect was “lost”. This is also evident from the gene-wise test graphs for these chromosome arms, as a set of *p*-values that is near zero for the ME test, but not for CN or joint tests (Additional file 2: Figure S8 and S16 for 8q and 6q, respectively).

#### Examples of effects found

Our results highlight a variety of effects of copy number and methylation explaining gene expression variability. To illustrate this, we selected probes found with our tests and examined corresponding patterns in the data motivating the findings. For each test, we selected probes for which a test had *p*-value ≤0.001. Specific criteria used to select mRNA probes for detailed analysis are given in subsection “Examples of genes found: selection criteria” of Additional file 2.

**Fig. 6:**
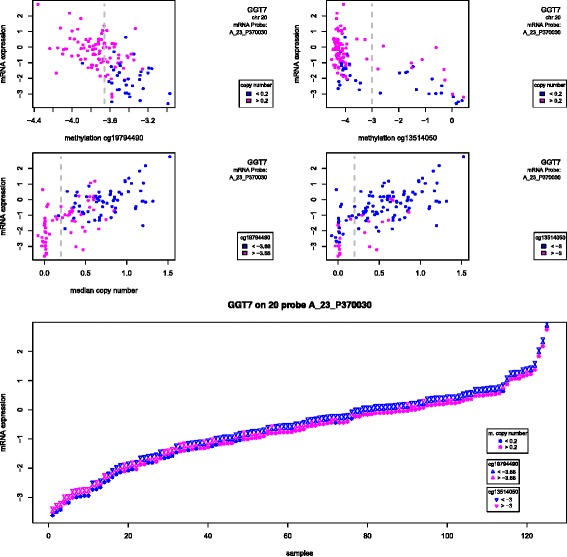
Gene *GGT7*: expression, methylation and copy number. mRNA expression (y-axis) of one probe mapping to gene *GGT7* in the colon cancer data. *Top* graphs: methylation values (logit of beta values) for chosen probes within 50 Kb of the gene start site; point colours represent the dychotomized copy number (see legend and dashed line in the graph right below), with blue corresponding to approximately diploid number of copies; the vertical dashed line represents the cut-off used to separate samples with more or less methylation. *Middle* graphs: median copy number values across all measurements within the 1 Mb region around the gene start site; point colours represent the dychotomized methylation (see legend and dashed line in the graph right above), with blue corresponding to less methylation; the vertical dashed line represents the cut-off used to separate samples approximately diploid from those. *Bottom* graph: mRNA expression for all samples, sorted from the smallest to the largest; three plotting symbols are used per sample to convey dychotomized copy number (star), methylation probe 1 (upwards triangle) and methylation probe 2 (downwards triangle); symbol colours are blue for lower values (diploid copy number, less methylation) or pink (copy gain, more methylation), as before

The selection criteria used are likely to find mRNA probes with strong copy number effects on 13q, 20q and 8q, as we know these characterize the colon cancer data. Indeed, this is what we found, but with an interesting twist: copy number seemed to explain mRNA expression for only part of the samples, with in almost all cases a subset of samples with diploid copy number and yet varying mRNA expression. This was the case with genes *GGT7, PIGU, NUFIP1, WFDC2, SLC39A4, PCDH20* and *IFT52* (Fig. 6 and Additional file 2: Figure S17 and S22). Since these probes were also selected on the basis of the ME test, we expected methylation to also regulate mRNA expression, in particular in diploid samples, in spite of the strong copy number effect. Indeed, we found that, not only samples with DNA copy gain also have less methylation, but also diploid samples more often display more methylation. For these samples, it seems that copy number and methylation have an additive effect on mRNA expression. Note, however, that in most cases, copy number and methylation did not explain mRNA expression completely, with a few samples displaying less methylation as well as a copy gain, and yet being under-expressed compared to the remaining samples. This suggests that a third mechanism is regulating mRNA expression for a subset of samples, for example down-regulating mRNA expression in samples with DNA copy gain and less than average methylation, or up-regulating mRNA expression in diploid samples with more than average methylation. In such cases, the event of up- or down-regulation of mRNA expression is thus achieved by different mechanisms depending on the sample, which illustrates the concept of complementary effects introduced in the simulation study.

It is interesting to note that the mRNA regulatory mechanism just described implies that DNA copy number and methylation are negatively correlated. This is the opposite from what we expect if a copy number change occurs at random, which would alter methylation in the same direction. This suggests again that the methylation changes are functional, since they neither can be a consequence of DNA copy gain nor are they likely to have occurred by chance (and thus not be functional) on the diploid samples.

**Fig. 7:**
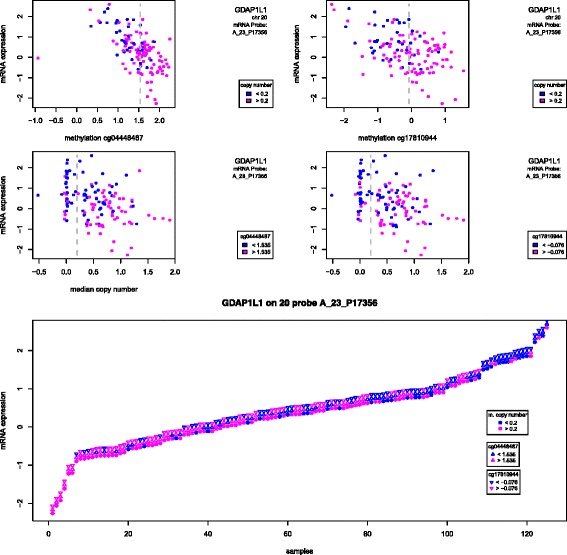
Gene *GDAP1L1*: expression, methylation and copy number. mRNA expression (y-axis) of one probe mapping to gene *GDAP1L1* in the colon cancer data. *Top* graphs: methylation values (logit of beta values) for chosen probes within 50Kb of the gene start site; point colours represent the dychotomized copy number (see legend and dashed line in the graph right below), with blue corresponding to approximately diploid number of copies; the vertical dashed line represents the cut-off used to separate samples with more or less methylation. *Middle* graphs: median copy number values across all measurements within the 1 Mb region around the gene start site; point colours represent the dychotomized methylation (see legend and dashed line in the graph right above), with blue corresponding to less methylation; the vertical dashed line represents the cut-off used to separate samples approximately diploid from those. *Bottom* graph: mRNA expression for all samples, sorted from the smallest to the largest; three plotting symbols are used per sample to convey dychotomized copy number (star), methylation probe 1 (upwards triangle) and methylation probe 2 (downwards triangle); symbol colours are blue for lower values (diploid copy number, less methylation) or pink (copy gain, more methylation), as before

Not all probes followed this pattern. Probe A_23_P17356, mapping to gene *GDAP1L1*, is interesting because copy number also separated samples into diploid and copy gain, but now copy gain samples are under-expressed compared with diploid ones (Fig. 7). Methylation still displayed a (negative) association with gene expression, for at least two probes. It is possible that methylation compensated for the DNA copy gain here, since 20q is very often gained in colon cancer.

Finally, the probe selected on 19q on the basis of the ME and joint tests, mapping to the *SLC7A9*, showed indeed methylation negatively correlated with mRNA expression (Additional file 2: Figure S23). As expected, DNA copy number was not correlated with mRNA expression.

The probe chosen mapping to *CDKN2A* displays a similar pattern to that for *SLC7A9*, with a trend of over-expression for samples displaying more methylation by one or both methylation probes. On the other hand, almost all samples with under-expression displayed more methylation with at least one of the two methylation probes (Additional file 2: Figure S24). This was not observed for the same probe in breast cancer samples. We conclude that expression of *CDKN2A* is often regulated via methylation, but that this can be best represented by different mRNA probes, for breast and colon cancer. Methylation probes showing strong association with mRNA expression may be the same, as is here the case.

After observing that methylation plays an important role in gene-expression regulation in breast cancer, we also looked for genes that had, for the ME test, *p*≤0.001 and *p*>0.1 in breast and colon cancer, respectively. This yielded 439 genes. In order to make the selection even stricter, we required that each gene had at least two mRNA probes satisfying the criterion, which led to 274 genes. This list included gene *MGC29506*, also known as *MZB1*. It has been previously found to be frequently methylated in hepatocellular cancer [14]. Of the 4 mRNA probes mapping to it, two are methylated above average for all samples (data not shown). Another gene on this list is *RAB40C*, a member of the RAS oncogene family. Of the 6 mRNA probes mapping to it, 2 are significant and exhibit a negative correlation between median copy number and mRNA expression (data not shown).

The above mechanisms illustrate effect types that can be found by our approach, squeezing relevant information out from thousands of mRNA probes, copy number and methylation measurements and yielding a list of mRNAs for further investigation. Obviously, correlation does not mean causation: further research would be needed to verify which of the associations found indeed is functional. In particular, statistical significance need not lead to biological relevance: as with any statistical method, there are effects found of too small a magnitude to be biologically relevant.

## Discussion

We have proposed a test to find associations between a dependent variable and two or more sets of covariates. It can be used for example to test for associations between a gene’s expression levels with copy number and/or methylation in a region around it. These help us better understand molecular mechanisms of gene expression regulation, individually and jointly, as we showed with the colon and breast cancer TCGA data.

In our illustrations, we have not corrected for multiple testing, even though many hypotheses were tested simultaneously. This was done for the sake of clarity: our illustrations involved comparing results from different data sets, and such comparisons are better made on the basis of uncorrected *p*-values. Indeed, multiple testing correction such as FDR may for example yield the same corrected *p*-value for many hypotheses, and it often does, even if uncorrected *p*-values are distinct. In practice, obviously multiple testing correction must be used if the test is applied many times.

Other methods have been proposed to look for associations between one molecular profile, and two or more other profiles. One framework that has been used by various authors for the two-dataset case (one independent) and, by some, has been extended to the three-dataset case, is that of penalized canonical correlations [5, 6, 15]. This framework tries to find (linear) combinations of the variables in the molecular profiles datasets that are (most) strongly correlated. As such, they are of an exploratory nature. Indeed, canonical vectors essentially identify sets of variables that are most strongly correlated. While their correlation may be considered high, it could still be due to chance, especially considering the high-dimensionality and sparseness of the data sets. In addition, due to their very nature sparse-canonical correlation-based approaches are computationally complex, and the complexity increases very quickly with each added independent dataset, as well illustrated by Lee et al. [15]. Compounded with the high-dimensions of the datasets involved, this means that such methods do not easily scale up. Furthermore, it can be difficult to interpret the canonical vectors.

In contrast, our approach is meant to be used when researchers have given sets of interest, which is is often the case in practice. Here we studied gene expression-regulating mechanisms by copy number and methylation, and used sets of measurements is *cis*, in and around the gene are of interest. We could have also considered *trans* effects by simply expanding the set. To study gene expression-regulating mechanisms by microRNA expression, one could form a set of microRNA (families) that may target that gene. To study eQTLs affecting a gene’s expression, natural sets are all SNPs in haploblocks mapping to a 2 Mb region centered around the start of the gene [16]. Our approach then tests the association of the dependent variable with all covariates in the set.

Bayesian approaches have also been proposed to analyse two datasets (one independent) together [17–19], but as such approaches are naturally computationally complex, they often become prohibitively computationally complex when used on the whole genome, or for more than one independent data set. Richardson et al. [18] tried to address the complexity by proposing a more efficient algorithm for MCMC estimation, although they do not apply their method to more than two data sets in their paper.

Our approach is computationally simple, so it can be scaled up to the whole genome and be used with many covariate sets. The simplicity results from the focus on testing, rather than on model fitting, as do Bayesian approaches, or on finding canonical vectors. This allows for quick, simple and objective evaluation of results, and easy prioritization of found associations.

Vaske et al. [20] proposed another method to analyse multiple molecular profiles simultaneously. Their method first replaces multiple probes by pathways, producing a matrix of inferred associations between samples and pathways, which is used, instead of the entire dataset, in analyses. This data with reduced dimensions is then used to build directed graphs. This process involves discretization of the data set, to enable establishment of direction in the graph estimation. As such, it relies on pathways containing information relevant to the study, as well as biological information about molecular interactions within pathways. This makes for a much more structured method, that is of particular interest when specific pathways and/or processes are under study. Indeed, results are reported per pathway, so relating them back to the genes is not trivial.

Our method is less structured, and this enables us to find a wide variety of types of association between covariates and the response which, as we saw in the TCGA data example, may not be linked to any known pathway. So it is preferred in cases where no particular effect is *a priori* of interest. The lack of imposed structure makes it more flexible. Indeed, our approach could be used on the matrix of inferred pathway activities generated by the approach of Vaske et al. [20], instead of their proposed graphical model, where we would have ignored the interaction factors. Here all pathways could be used in a single set of covariates to study relationship with a clinical outcome, for example. In particular, results are easy to interpret: not only it indicates clearly if the response is associated with the covariates, but also we can decompose each test statistic into the separate contributions of each covariate towards the joint set, pinpointing covariates driving the test result [7].

In the TCGA data examples, we observed similarities and differences between how copy number and methylation regulate gene expression in these two cancer types. For some chromosome arms, such as 1q and 17q, proportions of discoveries are similar between the two cancers. This needs not mean that the same gene expression-regulating mechanisms are involved, but that genomic changes of the same type are involved at the same ratio. Also, copy number and methylation do not necessarily explain gene expression variation of the same genes, and the joint test finds additional associations compared to the individual tests. For other arms, such as 13q and 20q, there are clear differences between the genomic changes found to affect gene expression in colon and breast cancer.

Some genes were selected to illustrate patterns found by the method, which showed some expression-regulating mechanisms driven by a combination of copy number and methylation changes. This is just the tip of the iceberg, with many other potentially interesting effects contained in the results. Researchers interested in further exploring our results can use the whole-genome results table provided (Additional files 3 and 4).

The pioneering articles of TCGA colon [21] and breast [22] cancer samples characterization have of course already given a detailed overview of molecular changes observed in these data sets. They have, however, not looked for complex regulatory mechanisms as we do here. So, our findings complement theirs, although we must be careful when extending the results from the samples subset used here to a larger set of cancer samples, as we cannot guarantee that the samples we chose represent well all similar cancer samples.

In the illustrations above we assumed that gene expression was the molecular phenotype of interest. Clearly that is not always the case, and other molecular phenotypes of interest include protein expression profiles. Our method can equally well be applied to such cases, with the reason for us not to have done so is the relatively limited number of samples with protein profiles available. In our experience, a minimum number of samples needed to yield reliable results is about 20 for the two-dataset case (one independent), but that of course is very much dependent on the signal-to-noise ratio in the data sets.

Our approach can also be used in a generalized-linear model context, where the dependent variables are related to the sets of covariates via a link function, such as the logarithm or the logit. This extension is straightforward, as we use the framework of the global test [7, 9]. This allows us for example to consider RNA-Seq data as dependent variables, if a suitable data transformation exists such that covariate effects can represent well variability in the transformed RNA-Seq data. Ideally, we would like to represent the data variability using the negative binomial, as done for example by the BioConductor packages edgeR ([23]) and DESeq ([24]), as it handles the data overdispersion ([25]). Note, however, that this is not straightforward as the negative binomial distribution is not in the exponential family. This extension is beyond the objectives of this paper and will appear elsewhere.

Another application of interest is to study the impact of changes in multiple molecular profiles on patients’ survival outcome, a variable formed by time from diagnosis to event and event information. This can be done by means of a Cox proportional-hazards model as an extension of the global test for this setting proposed earlier [26].

## Conclusions

We propose a method to find complex associations between multiple covariate sets, for example molecular profiles, and a response, which can be a single clinical variable or part of a molecular profile. Computationally efficient and flexible, our method can help unravel complex gene expression-regulating mechanisms.

## Additional files

Additional file 1
**Details in development of the test statistic.** This.pdf file includes details on how the test statistic is obtained, including a general functional form for the test statistic for any number *M* of covariates set and the expression obtained for *M*=3. (PDF 221 kb)

Additional file 2
**Supplementary tables and figures.** This.pdf file contains details about the simulation study setup, as well as all supplementary figures and tables. (PDF 3,401 kb)

Additional file 3
**Table of all results for the colon cancer data set.** (TXT 5,710 kb)

Additional file 4
**Table of all results for the breast cancer data set.** (TXT 5,742 kb)
