# Supplementary material for: Analysing multiple types of molecular profiles simultaneously: connecting the needles in the haystack
Source: BMC Bioinformatics. 2016 Feb 9;17:77. doi: 10.1186/s12859-016-0926-8 (PMC4746904; doi:10.1186/s12859-016-0926-8)
Supplement: Additional file 1 — Details in development of the test statistic. This.pdf file includes details on how the test statistic is obtained, including a general functional form for the test statistic for any number M of covariates set and the expression obtained for M=3. (PDF 221 kb) [file 12859_2016_926_MOESM1_ESM.pdf]

December 10, 2015

Details in development of test statistic

Additional File 1 to: Analysing multiple types of molecular profiles simultaneously: connecting the needles in the haystack

Menezes RX, Mohammadi L, Goeman JJ and Boer JM

1 Introduction

The global test statistic proposed by Goeman *et al.* [1] for testing associations between a response and a set of covariates used a test statistic proposed elsewhere [2], for testing in the context of random effects models. In [2] the authors get to their test statistic by first obtaining an approximation of the likelihood function using a truncated Taylor series expansion, and then computing the score test statistic. The advantage this yielded was partly due to the fact that the approximation was calculated around points where the random effects r_i were equal to zero. Since under H_0 it was assumed that $E(r_i) = 0$, some simplification followed.

Here we will follow on the footsteps of Le Cessie and van Houwelingen [2] to obtain a generalization of the global test statistic, for the case where two sets of covariates are involved. We will later on show that an extension to considering any fixed number of sets of covariates is straightforward.

2 Generalizaton: the global test for two sets of covariates

Consider the model

$$\mathrm{E}(Y_i) = h^{-1}\left(\alpha + r_i\right), \quad i = 1, \ldots, I,$$

for I samples, where the random effect r_i is here defined as a sum of random effects

$$r_i = X_i\boldsymbol{\beta} + Z_i\boldsymbol{\gamma}, \quad i = 1, \ldots, I, \qquad \mathbf{r} = (r_1, \ldots, r_I)^t,$$

with $\mathrm{E}(\boldsymbol{\beta}) = \mathrm{E}(\boldsymbol{\gamma}) = 0$, $\mathrm{Cov}(\boldsymbol{\beta}) = \sigma^2 I$, $\mathrm{Cov}(\boldsymbol{\gamma}) = \tau^2 I$ and $\mathrm{Cov}(\boldsymbol{\beta}, \boldsymbol{\gamma}) = 0$. These assumptions imply that $\mathrm{E}(r_i) = 0$ and $\mathrm{Cov}(r_i) = \sigma^2 R + \tau^2 S$, where we define $R \equiv XX^t$, $S \equiv ZZ^t$, and X, Z as being the matrices with the ith row represented by X_i, Z_i. Note that this means $\boldsymbol{\beta}, \boldsymbol{\gamma}$ are $p \times 1, q \times 1$ vectors and X, Z are $n \times p, n \times q$ matrices, respectively.

The likelihood function for all $\{Y_i\}$ can be written as

$$L(\boldsymbol{\beta}, \boldsymbol{\gamma}, \sigma, \tau) = \mathrm{E}_r \left[\prod_{i=1}^{I} f_i \left(Y_i | r_i, \boldsymbol{\beta}, \boldsymbol{\gamma}, \sigma, \tau \right) \right], \tag{1}$$

with f_i representing the density of Y_i, given the random effect r_i. Le Cessie and van Houwelingen [2] obtained the following approximation:

$$\begin{aligned} \prod_{i=1}^{I} f_i(r_i) = & \prod_{i=1}^{I} f_i(0) \Big\{ 1 + \sum_{i=1}^{I} \left[r_i u_i^1(0) + r_i^2 \left((u_i^1(0))^2 + u_i^2(0) \right) /2 \right] \\ + & \ (1/2) \sum_{i,j \neq i} r_i r_j u_i^1(0) u_j^1(0) \Big\} + o(\mathbf{r}^2), \end{aligned} \tag{2}$$

where $u_i^k(r_i) \equiv \partial^k \log f_i(r_i) / \partial r_i^k$, for $k = 1, 2, \ldots$. The expected value of (2) in r leads to an approximation to the likelihood (1), which can be written as

$$\begin{aligned} & \left[\prod_{i=1}^{I} f_i(0) \right] \Big\{ 1 + \frac{1}{2} \sum_{i=1}^{I} \left(\sigma^2 R_{ii} + \tau^2 S_{ii} \right) u_i^2(0) \\ & + \frac{1}{2} \sum_{i=1}^{I} \sum_{j=1}^{I} \left(\sigma^2 R_{ij} + \tau^2 S_{ij} \right) u_i^1(0) u_j^1(0) \Big\} + o(\sigma^2) + o(\tau^2). \end{aligned} \tag{3}$$

For simplicity, let us represent $\boldsymbol{\theta} = (\theta_1, \theta_2) \equiv (\sigma, \tau)$. We would like to test the hypotheses

$$H_0 : \boldsymbol{\theta} = (0,0) \equiv \boldsymbol{\theta}_0 \text{ against } H_a : \theta_j > 0, \text{ for at least one } j, \ j = 1, 2,$$

for at least one j, $j = 1, 2$. Such a test statistic can be derived for example from the inner product of the score vector

$$\mathbf{U}\left(\boldsymbol{\theta}_0\right)^t \mathbf{U}\left(\boldsymbol{\theta}_0\right), \tag{4}$$

where $\mathbf{U}\left(\boldsymbol{\theta}_0\right)$ represents the score vector, given by

$$\mathbf{U}\left(\boldsymbol{\theta}_0\right) = \left[\frac{\partial}{\partial \sigma^2} \log L(\boldsymbol{\beta}, \boldsymbol{\gamma}, \boldsymbol{\theta}) \Big|_{H_0}, \frac{\partial}{\partial \tau^2} \log L(\boldsymbol{\beta}, \boldsymbol{\gamma}, \boldsymbol{\theta}) \Big|_{H_0} \right].$$

From (3) we have that, under H_0, the likelihood is reduced to

$$L(\boldsymbol{\beta}, \boldsymbol{\gamma}, \boldsymbol{\theta})|_{H_0} = \prod_{i=1}^{I} f_i(0).$$

Also using (3) we can write

$$\frac{\partial}{\partial \sigma^2} \log L(\boldsymbol{\beta}, \boldsymbol{\gamma}, \boldsymbol{\theta})\bigg|_{H_0} = \sum_{i=1}^{I} R_{ii} u_i^2(0) + \sum_{i=1}^{I}\sum_{j=1}^{I} R_{ij} u_i^1(0) u_j^1(0). \tag{5}$$

If we assume that $Y_i \sim \mathcal{G}$ where $\mathcal{G}$ is a distribution in the exponential family with canonical link, we can write its density function as

$$f(y; \delta, \phi) = \exp\left\{[y\delta - b(\delta)]/a(\phi) + c(y, \phi)\right\},$$

where δ represents the parameter of interest and ϕ is a known nuisance parameter, with $a(.)$ known. Then

$$\begin{aligned} \frac{\partial}{\partial r_i} \log f(y; \delta, \phi) &= \frac{y_i - \mu_{1i}(r_i)}{a(\phi)} \equiv u_i^1(r_i) \\ \frac{\partial^2}{\partial r_i^2} \log f(y; \delta, \phi) &= \frac{-\mu_{2i}(r_i)}{a(\phi)^2} \equiv u_i^2(r_i), \end{aligned}$$

where $\mu_{1i}(r_i) = \partial b(\delta)/\partial r_i$ and $\mu_{2i}(r_i) = a(\phi)\partial^2 b(\delta)/\partial r_i^2$. Replacing these expressions into (5), we can write

$$\begin{aligned} \frac{\partial}{\partial \sigma^2} \log L(\boldsymbol{\beta}, \boldsymbol{\gamma}, \boldsymbol{\theta})\bigg|_{H_0} &= \frac{1}{2a(\phi)^2}\Big\{(Y - \mu_1)^t R (Y - \mu_1) - \mathrm{tr}(RV)\Big\} \\ &\equiv \frac{1}{2a(\phi)^2}\left\{Q(R) - \mathrm{tr}(RV)\right\}, \end{aligned} \tag{6}$$

where we define $Q(A) \equiv (Y - \mu_1)^t A (Y - \mu_1)$ for a square matrix A with as many rows as the number I of entries in Y, $V = \mathrm{diag}\{\mu_{2i}(r_i)\}$ and μ_1 is a $I \times 1$ vector with ith entry given by μ_{1i}. Following the same steps, we obtain a similar expression for the partial derivative with respect to τ^2:

$$\begin{aligned} \frac{\partial}{\partial \tau^2} \log L(\boldsymbol{\beta}, \boldsymbol{\gamma}, \boldsymbol{\theta})\bigg|_{H_0} &= \frac{1}{2a(\phi)^2}\Big\{(Y - \mu_1)^t S (Y - \mu_1) - \mathrm{tr}(SV)\Big\} \\ &\equiv \frac{1}{2a(\phi)^2}\left\{Q(S) - \mathrm{tr}(SV)\right\}, \end{aligned} \tag{7}$$

where here $Q(S) \equiv (Y - \mu_1)^t S (Y - \mu_1)$.

Note that the expressions (6,7) are the entries in $\mathbf{U}(\boldsymbol{\theta})$. We have $\mathrm{E}[Q(R)] = \mathrm{tr}(RV)$ and, equivalently, $\mathrm{E}[Q(S)] = \mathrm{tr}(SV)$. Replacing this in (6) and (7), we can express the inner product of the score vector as

$$\left\{Q(R) - \mathrm{E}[Q(R)]\right\}^2 + \left\{Q(S) - \mathrm{E}[Q(S)]\right\}^2,$$

up to a constant. This is the test statistic sought.

3 Considering any K number of sets of covariates

The extension to considering an arbitrary number K of sets of covariates is relatively straightforward, as we will show below[1]. Consider the model

$$\mathrm{E}(Y_i) = h^{-1}(\alpha + r_i), \quad i = 1, \ldots, I,$$

where the random effect r_i is here defined as a sum of random effects

$$r_i = \sum_{k=1}^{K} X_{ki}\boldsymbol{\beta}_k, \quad i = 1, \ldots, I, \qquad \mathbf{r} = (r_1, \ldots, r_I)^t,$$

with $\mathrm{E}(\boldsymbol{\beta}_k) = 0$, $\mathrm{Cov}(\boldsymbol{\beta}_k) = \sigma_k^2 I$ and $\mathrm{Cov}(\boldsymbol{\beta}_{k1}, \boldsymbol{\beta}_{k2}) = 0$ for $k = 1, \ldots, K,\ k_1 \neq k_2$. These assumptions have similar implications to the 2-gene set case, namely that $\mathrm{E}(r_i) = 0$ and $\mathrm{Cov}(r_i) = \sum_{k=1}^{K} \sigma_k^2 R_k$, where we now define $R_k \equiv X_k X_k^t$, with X_k representing the matrix which ith row is represented by X_{ki}.

The likelihood function for all $\{Y_i\}$ can be written as

$$L(\boldsymbol{\beta}, \boldsymbol{\gamma}, \sigma, \tau) = \mathrm{E}_r \left[\prod_{i=1}^{I} f_i \left(Y_i | r_i, \{\boldsymbol{\beta}_k\}, \{\sigma_k\} \right) \right], \tag{8}$$

with f_i representing the density of Y_i, given the random effect r_i. Here $\prod_i f_i(r_i)$ can be approximated by (2) as before, and taking the expected value in r now leads to

$$\left[\prod_{i=1}^{I} f_i(0) \right] \Big\{ 1 + \frac{1}{2} \sum_{i=1}^{I} u_i^2(0) \sum_{k=1}^{K} \sigma_k^2 R_{k,ii} + \frac{1}{2} \sum_{i=1}^{I} \sum_{j=1}^{I} u_i^1(0) u_j^1(0) \sum_{k=1}^{K} \sigma_k^2 R_{k,ij} \Big\} + \sum_{k=1}^{K} o(\sigma_k^2). \tag{9}$$

Here we shall define $\boldsymbol{\theta} = (\sigma_1, \sigma_2, \ldots, \sigma_K)$. The kth entry of the score vector is now

$$\left. \frac{\partial}{\partial \sigma_k^2} \log L(\{\boldsymbol{\beta}_k\}, \boldsymbol{\theta}) \right|_{H_0}$$

Under H_0, the likelihood is again reduced to $\prod_i f_i(0)$. In addition, similarly to (5) we now have

$$\left. \frac{\partial}{\partial \sigma_k^2} \log L(\{\boldsymbol{\beta}_k\}, \boldsymbol{\theta}) \right|_{H_0} = \sum_{i=1}^{I} R_{k,ii} u_i^2(0) + \sum_{i=1}^{I} \sum_{j=1}^{I} R_{k,ij} u_i^1(0) u_j^1(0). \tag{10}$$

[1]Note that in the main text we have represented the number of covariate sets by M.

By using again the fact that Y_i has a distribution $\mathcal{G}$ in the exponential family with canonical link, we get that the kth entry of the score vector $\mathbf{U}(\boldsymbol{\theta})$ is

$$\frac{\partial}{\partial \sigma_k^2} \log L(\{\boldsymbol{\beta}_k\}, \boldsymbol{\theta})\Big|_{H_0} = \frac{1}{2a(\phi)^2} \Big\{ (Y-\mu_1)^t R_k (Y-\mu_1) - \mathrm{tr}(R_k V) \Big\}$$
$$\equiv \frac{1}{2a(\phi)^2} \{ Q(R_k) - \mathrm{tr}(R_k V) \}, \quad k = 1, \ldots, K. \quad (11)$$

So, the inner product of the score vector is given by, up to a constant,

$$\sum_{k=1}^{K} \{ Q(R_k) - \mathrm{tr}(R_k V) \}^2 \equiv \sum_{k=1}^{K} \{ Q(R_k) - \mathrm{E}(R_k) \}^2 , \quad (12)$$

which can then be used to test

$$H_0 : \boldsymbol{\theta} = (0, \ldots, 0) \equiv \boldsymbol{\theta}_0 \text{ against } H_a : \theta_k > 0, \text{ for at least one } k, \; k = 1, \ldots, K.$$

4 Alternative scaling of the test statistics

In the Methods section of the main article we suggest using the scaled individual test statistics, in order to combine effects of individual covariate sets onto the same scale on the joint test statistic. Here we give an alternative to using the scaled test statistics, by scaling and centering the covariates prior to computing the individual test statistics $Q(X), Q(Z)$ and subsequently computing $Q(X,Z) \equiv Q(X) + Q(Z)$. Indeed, we could compute directly $Q(X_S)$ and $Q(Z_S)$, where $X_S \equiv Xw_X = X(I - H_X)/\ \mathrm{trace}(XX^t)$ and $Z_S \equiv Zw_Z = (I - H_Z)/\ \mathrm{trace}(XX^t)$. That is, we suggest using

$$Q(X,Z) = Q(Xw_X) + Q(Zw_Z) = \frac{(Y-\mu)^t \left[Xw_X^2 X^t + Zw_Z^2 Z^t \right] (Y-\mu)}{(Y-\mu)^t (Y-\mu)}, \quad (13)$$

where $w_X = [\ \mathrm{trace}(XX^t)(I - H_X)]^{-1}$ and $I - H_X$ is the matrix that centers X around its column-specific means, with a similar definition for w_Z. Since $E[Q(X)] =\ \mathrm{trace}(XX^t)/J$ [1], the proposed weights correspond to bringing mean and variance of $Q(X), Q(Z)$ onto the same scale.

References

[1] Goeman J, van de Geer S, de Kort F, van Houwelingen H: **A global test for groups of genes: testing association with a clinical outcome**. *Bioinformatics* 2004, **20**:93–99.

[2] Le Cessie S, van Houwelingen H: **Testing the Fit of A Regression-Model Via Score Tests in Random Effects Models**. *Biometrics* 1995, **51**:600–614.
