# Supplementary material for: Analysing multiple types of molecular profiles simultaneously: connecting the needles in the haystack
Source: BMC Bioinformatics. 2016 Feb 9;17:77. doi: 10.1186/s12859-016-0926-8 (PMC4746904; doi:10.1186/s12859-016-0926-8)
Supplement: Additional file 2 — Supplementary tables and figures. This.pdf file contains details about the simulation study setup, as well as all supplementary figures and tables. (PDF 3,401 kb) [file 12859_2016_926_MOESM2_ESM.pdf]

December 14, 2015

Details of simulation study setup, supplementary tables and figures

Additional File 2 to: Analysing multiple types of molecular profiles simultaneously: connecting the needles in the haystack

Menezes RX, Mohammadi L, Goeman JJ and Boer JM

1 Simulation study setup

1.1 Basic structure

Here we describe in detail the setup used for our simulation study, presented in the Results section of the main article. Our objective with this simulation study is to evaluate power to detect various types of effects, using the proposed test statistics.

We consider four independent data sets, each involving one set of variables $\{Y_i, X_j, Z_k\}$, with $i, = 1, \ldots, 1000$ and $j = 1, \ldots, J,\ ,\ k = 1, \ldots, K$. Each data set can be seen as a (genomic) region, here assumed to involve one association type between the covariate sets and the dependent variable Y_i for $i = 1, \ldots, 500$, and no association for the remaining probes. The associations considered are: region I, where Y_i is associated with $\{X_j\}$ only, which we will refer to as "x only"; region II, "additive", where both covariate sets affect outcome linearly; region III, "multiplicative", where both covariate sets affect outcome linearly as well as multiplicatively; and region IV, "split-samples" or "complementary", where $\{Y_i\}$ depends upon $\{X_j\}$ for half of the samples, and for the other half $\{Y_i\}$ depends upon $\{Z_k\}$. For each data set, three sample sizes are considered: 50, 100 and 200 samples.

Within each region, we test for association between each Y_i and the covariate sets $\{X_j\}, \{Z_k\}$ using the test statistic $Q(X, Z)$ proposed in the Methods section of the main text. Here p-values are estimated by comparing the observed test statistic to values obtained after permuting the dependent variable samples 1000 times, and re-computing the test statistics. For the aims of this particular study, which is to compare ROC curves, no multiple testing is necessary.

Then we take

$$Y_i = f_{X,Z,i}\left(\{X_j,\ j = 1, \ldots, J\}, \{Z_k,\ k = 1, \ldots, K\}\right) + V_i,$$

where $V_i \sim \mathcal{N}(0.1, 0.5)$, independently of the other covariates, and $f_{X,Z,i}$ is a given function that determines how the covariates affect the dependent variable. Effects are assumed to be in *cis*, and specifically we define that only X_{i-1}, X_i, X_{i+1} and Z_{i-1}, Z_i, Z_{i+1} are involved in $f_{X,Z,i}$ for each i.

Per region, we assume $f_{X,Z,i} \equiv f_{X,Z},\ i = 1, \ldots, I/2 = 500$, and $f_{X,Z,i} = 0,\ i = I/2 + 1, \ldots, I$.

All data above is generated for sample sizes of 50, 100 and 200. Thus a total of 12 data sets are generated, for all possible combinations of sample sizes and regions, and each data set consists of 3 data matrices, Y, X, Z, each with as many columns as samples.

This basic setup can then be used to simulate data, once we define how $\{X_j\}$ and $\{Z_k\}$ will be generated. We wish to introduce correlation between these covariates, and this is done in two ways: first we take Z_k as a function of X_k, for each k; subsequently, we generate $\{X_j\}$ and $\{Z_k\}$ according to a multivariate normal distribution with an empirical covariance matrix. These two approaches are explained in detail in the following subsections.

1.2 Z is a function of X

In this simulation study, correlation between X and Z is included by considering that Z is a function of X. Specifically, we assume that $X_j \sim \mathcal{N}(1, 2.25)$ for all j, with all X_j independent, and $Z_k \equiv X_k + W_k$, with $W_k \sim \mathcal{N}(0, \gamma)$, and $W_k \perp X_k$, for all k. For simplicity we assume that $I = J = K$. The relationship between X_j and Z_k implies that

$$\mathrm{E}(Z_k) = \mathrm{E}[\mathrm{E}(Z_k|X_k)] = \mathrm{E}(X_k) = 1,$$

and, similarly,

$$\mathrm{Var}(Z_k) = \mathrm{E}[\mathrm{Var}(Z_k|X_k)] + \mathrm{Var}[\mathrm{E}(Z_k|X_k)] = \mathrm{Var}(X_k) + \mathrm{Var}(W_k),$$

leading to $\mathrm{Cor}(X_k, Z_k) = \{\mathrm{Var}(X_k)/[\mathrm{Var}(X_k) + \mathrm{Var}(W_k)]\}^{1/2}$. So by changing $\mathrm{Var}(W_k)$, we can vary the correlation between X_k and Z_k. For example, if $\mathrm{Var}(W_k) = 1$, we get $\mathrm{Cor}(X_k, Z_k) = 0.83$ for all k, although observed values may vary due to randomness. On the other hand, if $\mathrm{Var}(W_k) = 5$, we get $\mathrm{Cor}(X_k, Z_k) = 0.56$. In all cases, $\mathrm{Cor}(X_j, Z_k) = 0$ for $j \neq k$.

We also wish to consider a case where X and Z are negatively correlated. For this, we will use the same setup and parameter values as above, with $\mathrm{Var}(W_k) = 1$, and define now $Z_k \equiv -X_k + W_k$. Note that the individual $\{X_j\}$ are uncorrelated, as are individual $\{Z_k\}$, in all cases presented above.

The definition of f per region is given in table 1.

1.3 Using empirical correlation structures

In practice, correlation structures can be considerably more complex than assumed in the previous subsection, so we here consider two empirical correlation matrices, derived from the TCGA colon cancer data studied in the subsection "Colon and breast cancer datasets". In this example, gene expression is explained by set of copy number (CN) or methylation (ME) covariates. Specifically, we chose genes *GGT7* and *GDAP1L1*, both on 20q. Both were found in

Table 1: Covariate effects on dependent variable Y_i, with Z a function of X

Region	Effect type	$f_{X,Z,i}$
I	X only	$g(X,i)$
II	Additive	$g(X,i) + g(Z,i)$
III	Multiplicative	$g(X,i) + g(Z,i) + 2 * g(X * Z, i)$
IV	Split samples-complementary	$3 * g(X,i)$ for half of the samples, $3 * g(Z,i)$ for remaining samples

$g(X,i) \equiv X_i + 0.5 * (X_{i-1} + X_{i+1})$

Table 2: Covariate effects on dependent variable Y_i, empirical correlation

Region	Effect type	$f_{X,Z,i}$
I	X only	$g(X,i)$
II	Additive	$g(X,i) + g(Z,i)$
III	Multiplicative	$(1/2) * [g(X,i) + g(Z,i) + g(X * Z, i)]$
IV	Split samples-complementary	$g(X,i)$ for half of the samples, $g(Z,i)$ for remaining samples

$g(X,i) \equiv [X_i + 0.5 * (X_{i-1} + X_{i+1})]/4$, $i = 1, \ldots, J$

our analysis to be associated with CN (within 1 Kb of its transcription start site) as well as ME (within 50 Kb of its transcription start site). For details of this analysis, see "Examples of effects found" in the main text.

For *GGT7*, tests involved 21 CN and 6 ME covariates. The majority of copy number and methylation covariates were negatively associated with each other, with the strongest Pearson correlation estimated as -0.83. Gene *GDAP1L1* was found to have expression associated with 20 CN and 2 ME covariates, but here CN and ME covariates are positively associated with each other, with Pearson correlation around 0.5. In both cases, covariates of the same type (CN or ME) are mostly positively associated, i.e., $\mathrm{Cor}(X_j, X_l) > 0, l \neq j$ for all pairs of CN variables, and $\mathrm{Cor}(Z_k, Z_l) > 0, l \neq k$ for most pairs of ME variables.

Using each correlation matrix, multivariate normal observations are generated for $\{X_j\}$ and $\{Z_k\}$, with $J = 21, K = 6$ and $J = 20, K = 2$ for *GGT7* and *GDAP1L1*, respectively. In order to make the means of $\{X_k\}$ and $\{Z_k\}$ resemble those of CN and ME covariates in relative terms, we use $\mathrm{E}(X_j) = 4$ and $\mathrm{E}(Z_k) = 2$ for all j, k. This lets copy number variables have an effect twice as large as that of methylation ones.

In this case, the number J, K of observed covariates $\{X_j\}, \{Z_k\}$ is smaller than the number I of responses $\{Y_i\}$. So we recycle X_j, Z_k to be able to generate all responses. Specifically, as given in table 2, $g(X,i)$ is a function of X_{i-1}, X_i, X_{i+1} for $i = 1, \ldots, J$. Then for $i = J+1, \ldots, 2J$ it is is a function of $X_{i-J-1}, X_{i-J}, X_{i_j+1}$, and this pattern is repeated for every J responses. A similar recycling rule is used for $\{Z_k\}$. So, we can write

$$g(U,i) = [U_l + 0.5 * (U_{l-1} + U_{l+1})]/4,$$

where l is equal to the remainder of i/J or i/K, if the remainder is positive, else $l = J$ or $l = K$, respectively for $U = X$ and $U = Z$. Here we define $X_0 = Z_0 = X_{J+1} = Z_{K+1} = 0$.

As a result, this setup involves not only correlation between the covariates, but also correlation between the responses.

1.4 Test statistics computed and significance

For each dependent variable Y_i, we test for its association with the covariate sets $\{X_j\}, \{Z_k\}$ using the test statistic given by the sum of the single set-test statistics $Q(X) + Q(Z)$. We use the non-standardized version of the test statistics, as it suffices to compute permutation-based p-values. We estimate p-values by permutation, using a total of 1000 permutations.

As the aim of this simulation study is to construct ROC curves for the test statistics under different types of effects, no multiple testing is here used.

1.5 ROC curves for simulation study where Z is a function of X

In the Results section of the main text, we present ROC curves for a simulated data set with 100 samples. Here we present ROC curves for sample sizes 50 (figure 1) and 200 (figure 2), which both corroborate the conclusions drawn with 100 samples.

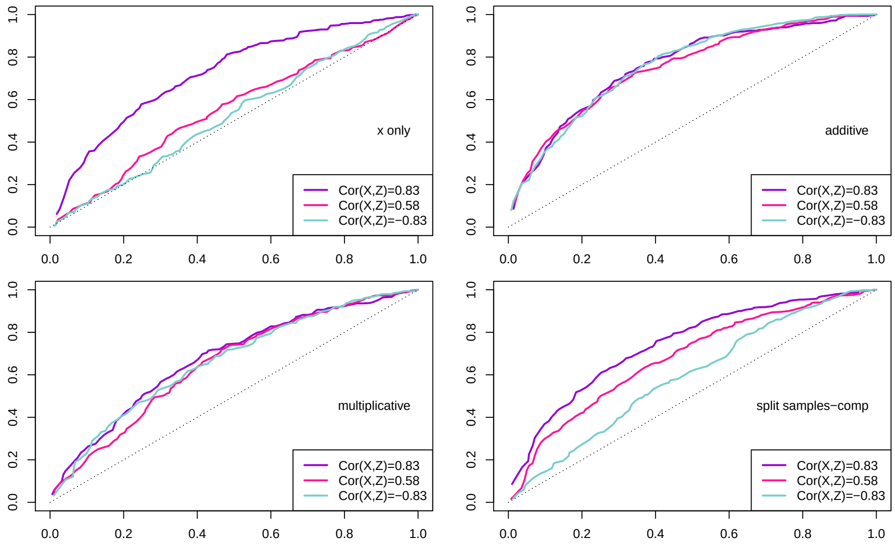

Figure 1: ROC curves to evaluate power to find four different types of effects between one dependent variable and two independent covariates sets, $N = 50$, Simulation Study with Z a function of X.

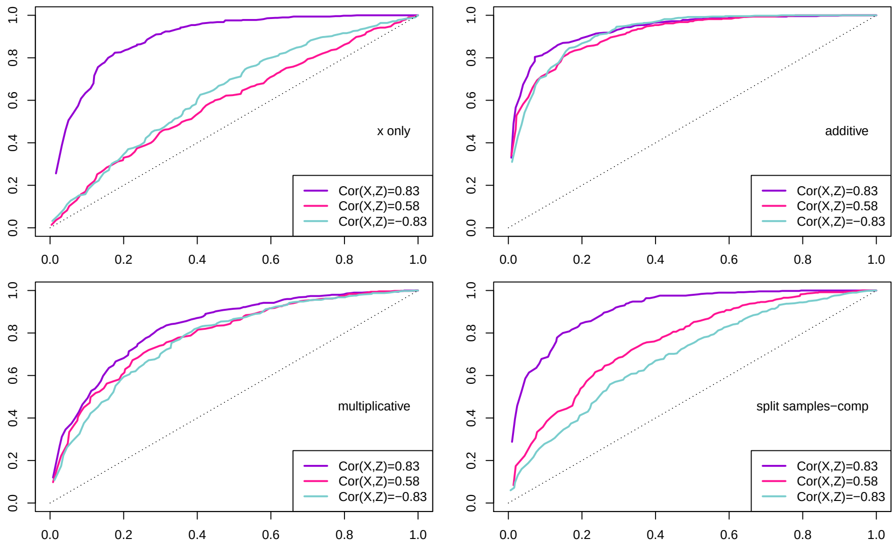

Figure 2: ROC curves to evaluate power to find four different types of effects between one dependent variable and two independent covariates sets, $N = 200$, Simulation Study with Z a function of X.

2 Analysis colon and breast cancer datasets

2.1 Details of data sets downloaded

We downloaded 125 colon and 173 breast cancer samples, which had been profiled for DNA copy number (CN), methylation (ME) and mRNA expression. DNA copy number data was derived from the Affymetrix SNP 6.0 microarray and had been segmented. We de-segmented it to produce individual intensities measured over 3×10^4 equally-spaced positions on the genome. The level-2 DNA-methylation profiles downloaded had been produced by the Infinium Human Methylation 27K array, which produces per probe a m and a u signal, representing methylated and unmethylated signals respectively. As measurement, we used the logit of the ratio $m/(m + u)$, to transform the ratios into real-valued variables as well as correct them for DNA dosage. This implies in particular that underlying copy number changes cannot influence the methylation measurements to the extend of leading to significant associations between mRNA and methylation. As such, associations between mRNA expression and methylation are independent of copy number changes.

The level-2 gene-expression profiles downloaded correspond to lowess-normalized log-ratios obtained using Agilent 450A arrays, involving 62335 probes with known genome location.

2.2 Examples of genes found: selection criteria

Firstly, we looked for mRNA probes selected with the joint test, as well as both CN and ME tests, on 13q, 20q and 8q, in the colon cancer data. We further refined our search by requiring that the probe had CN test p-value > 0.1 in the breast cancer data. This led to 56, 104 and 54 mRNA probes being selected, respectively on 13q, 20q and 8q. Secondly, we looked for probes that had both ME test and joint test p-values selected, on 19q in the colon cancer data. We again further refined the selection by selecting only probes that had ME test p-value > 0.1 in the breast cancer data. This led to 98 mRNA expression probes. From each one of these four lists, we selected a single probe to illustrate effects found, except for 13q and 20q from which we examined two and four probes, respectively. Finally, we considered also one probe mapping to the gene *CDKN2A*, for which p-values were just above cut-off for ME and joint tests, in colon cancer. A list of the probes selected, with annotation and tests results in both data sets, can be found in supplementary table 9.

2.3 Tables

	Colon		Breast	
chr	p	q	p	q
1	4098	3587	4092	3583
2	2065	3322	2064	3318
3	1994	2367	1992	2365
4	968	2256	968	2252
5	775	3068	773	3063
6	2375	1757	2370	1749
7	1321	2654	1319	2650
8	1139	1868	1138	1861
9	905	2172	904	2169
10	741	2277	740	2276
11	1504	2933	1499	2930
12	1089	2937	1088	2935
13	0	1368	0	1365
14	0	2496	0	2496
15	0	2483	0	2481
16	1761	1500	1760	1502
17	1164	2991	1159	2985
18	348	878	348	878
19	2009	2049	2009	2046
20	680	1283	679	1282
21	0	892	0	891
22	0	1777	0	1775

Table 3: Number of joint tests per chromosome and arm, per data set. Note that the number of tests of methylation effect only on mRNA expression may be smaller due to the fact that some mRNA probes have no methylation probes within 50Kb of the corresponding genes starting site.

ratio label	definition
copy number (CN)	$\#(Q(X) \leq \alpha)/$ total number of tests
methylation (ME)	$\#(Q(Z) \leq \alpha)/$ total number of tests
CN + ME (joint)	$\#(Q(X,Z) \leq \alpha)/$ total number of tests
CN and joint overlap	$\#[(Q(X) \leq \alpha)\&(Q(X,Z) \leq \alpha)]$ $/\#(Q(X,Z) \leq \alpha)$
ME and joint overlap	$\#[(Q(Z) \leq \alpha)\&(Q(X,Z) \leq \alpha)]$ $/\#(Q(X,Z) \leq \alpha)$
Joint sel but not CN ME/joint sel	$\#[(Q(X,Z) \leq \alpha)\&(Q(X) > \alpha)\&(Q(Z) > \alpha)]$ $/\#(Q(X,Z) \leq \alpha)$
CN sel but not joint/CN sel	$\#[(Q(X) \leq \alpha)\&(Q(X,Z) > \alpha)]$ $/\#(Q(X) \leq \alpha)$
ME sel but not joint/ME sel	$\#[(Q(Z) \leq \alpha)\&(Q(X,Z) > \alpha)]$ $/\#(Q(Z) \leq \alpha)$
(CN sel & ME sign)/CN sel	$\#[(Q(X) \leq \alpha)\&(Q(Z) \leq \alpha)]/$ $/\#(Q(X) \leq \alpha)$
(CN sel & ME sign)/ME sel	$\#[(Q(X) \leq \alpha)\&(Q(Z) \leq \alpha)]/$ $/\#(Q(Z) \leq \alpha)$

Table 4: Definitions of ratios computed using the test statistics.

	colon	breast
copy number (CN)	0.161 (73851)	0.080 (73754)
methylation (ME)	0.054 (51912)	0.051 (51852)
CN + ME (joint)	0.157 (73851)	0.090 (73754)
CN and joint overlap	0.801	0.698
ME and joint overlap	0.289	0.412
Joint sel but not CN ME/joint sel	0.006	0.012
CN sel but not joint/CN sel	0.218	0.209
ME sel but not joint/ME sel	0.154	0.266

Table 5: Proportions (total) of selected tests found for the colon and breast cancer data sets. Entries in the table are proportions, computed using definitions found in in supplementary table 2. Testing for association with methylation is not possible for all gene expression probes as some do not have any methylation probes within 50Kb of the gene´s transcription start site.

	CN and joint overlap	CN sel but not joint /CN sel	ME and joint overlap	ME sel but not joint /ME sel	CN	ME	CN + ME (joint)	Joint sel but not CN ME/ joint sel
1q	0.654	0.271	0.393	0.156	0.127	0.066	0.142	0.006
2q	0.623	0.418	0.434	0.078	0.079	0.035	0.073	0.004
3q	0.726	0.269	0.326	0.111	0.113	0.042	0.114	0.000
4q	0.872	0.192	0.176	0.239	0.139	0.030	0.129	0.000
5q	0.817	0.230	0.224	0.161	0.160	0.040	0.151	0.006
6q	0.855	0.173	0.183	0.302	0.142	0.036	0.137	0.008
7q	0.836	0.266	0.285	0.135	0.164	0.047	0.144	0.010
8q	0.951	0.061	0.272	0.179	0.201	0.066	0.199	0.003
9q	0.792	0.275	0.251	0.106	0.186	0.048	0.170	0.008
10q	0.753	0.247	0.299	0.147	0.119	0.042	0.119	0.004
11q	0.616	0.434	0.449	0.122	0.137	0.064	0.126	0.005
12q	0.767	0.260	0.297	0.126	0.165	0.054	0.159	0.009
13q	0.962	0.048	0.279	0.114	0.289	0.090	0.286	0.000
14q	0.958	0.084	0.124	0.273	0.296	0.048	0.284	0.004
15q	0.886	0.111	0.161	0.220	0.229	0.048	0.230	0.011
16q	0.680	0.398	0.440	0.093	0.151	0.065	0.133	0.010
17q	0.691	0.194	0.348	0.246	0.131	0.071	0.153	0.009
18q	0.982	0.125	0.114	0.161	0.292	0.035	0.26	0.004
19q	0.297	0.571	0.761	0.012	0.075	0.083	0.108	0.009
20q	0.971	0.053	0.349	0.102	0.383	0.145	0.373	0.008
21q	0.824	0.125	0.229	0.220	0.179	0.056	0.191	0.000
22q	0.746	0.248	0.289	0.219	0.159	0.059	0.160	0.004
1p	0.829	0.190	0.234	0.179	0.172	0.048	0.168	0.015
2p	0.431	0.491	0.599	0.012	0.056	0.040	0.066	0.022
3p	0.785	0.313	0.312	0.155	0.160	0.052	0.140	0.007
4p	0.909	0.168	0.171	0.263	0.185	0.039	0.169	0.000
5p	0.869	0.141	0.226	0.296	0.110	0.035	0.108	0.000
6p	0.572	0.319	0.464	0.165	0.088	0.059	0.105	0.008
7p	0.903	0.167	0.177	0.254	0.204	0.045	0.188	0.004
8p	0.939	0.061	0.218	0.306	0.173	0.054	0.173	0.005
9p	0.790	0.329	0.234	0.094	0.161	0.035	0.137	0.000
10p	0.831	0.316	0.215	0.300	0.107	0.027	0.088	0.000
11p	0.554	0.497	0.515	0.082	0.095	0.049	0.086	0.000
12p	0.783	0.245	0.280	0.214	0.150	0.051	0.144	0.000
16p	0.756	0.323	0.392	0.109	0.158	0.062	0.142	0.008
17p	0.968	0.045	0.132	0.293	0.270	0.050	0.266	0.000
18p	0.989	0.142	0.141	0.071	0.305	0.040	0.264	0.000
19p	0.593	0.475	0.439	0.061	0.157	0.065	0.139	0.021
20p	0.960	0.026	0.242	0.127	0.287	0.081	0.291	0.005

Table 6: Ratios of selected tests for the colon cancer data set, per chromosome arm. Definitions used are in supplementary table 2.

	CN and joint overlap	CN sel but not joint /CN sel	ME and joint overlap	ME sel but not joint /ME sel	CN	ME	CN + ME (joint)	Joint sel but not CN ME/ joint sel
1q	0.665	0.102	0.451	0.241	0.093	0.074	0.125	0.004
2q	0.552	0.352	0.514	0.156	0.054	0.039	0.063	0.010
3q	0.597	0.291	0.453	0.314	0.057	0.044	0.067	0.013
4q	0.683	0.330	0.366	0.422	0.046	0.028	0.045	0.000
5q	0.936	0.202	0.196	0.144	0.209	0.041	0.178	0.015
6q	0.826	0.237	0.209	0.526	0.053	0.022	0.049	0.023
7q	0.706	0.235	0.339	0.384	0.063	0.037	0.068	0.022
8q	0.917	0.089	0.199	0.551	0.084	0.037	0.084	0.019
9q	0.515	0.343	0.508	0.250	0.047	0.041	0.060	0.015
10q	0.623	0.253	0.421	0.226	0.042	0.027	0.050	0.000
11q	0.639	0.131	0.490	0.271	0.086	0.078	0.116	0.012
12q	0.650	0.310	0.459	0.175	0.079	0.047	0.084	0.012
13q	0.882	0.196	0.294	0.375	0.082	0.035	0.075	0.000
14q	0.751	0.211	0.354	0.309	0.072	0.039	0.076	0.016
15q	0.775	0.132	0.284	0.376	0.073	0.037	0.082	0.010
16q	0.880	0.153	0.327	0.218	0.196	0.079	0.189	0.011
17q	0.782	0.089	0.370	0.435	0.102	0.078	0.119	0.017
18q	0.800	0.077	0.300	0.182	0.059	0.025	0.068	0.017
19q	0.366	0.435	0.708	0.083	0.064	0.076	0.099	0.000
20q	0.722	0.140	0.424	0.291	0.094	0.067	0.112	0.007
21q	0.486	0.244	0.557	0.204	0.051	0.055	0.079	0.000
22q	0.763	0.116	0.329	0.368	0.106	0.064	0.123	0.018
1p	0.637	0.221	0.464	0.256	0.075	0.057	0.092	0.021
2p	0.710	0.344	0.393	0.219	0.076	0.035	0.070	0.007
3p	0.721	0.255	0.389	0.221	0.092	0.048	0.095	0.000
4p	0.802	0.170	0.418	0.191	0.091	0.049	0.094	0.000
5p	0.455	0.474	0.591	0.435	0.025	0.030	0.028	0.000
6p	0.558	0.354	0.515	0.159	0.075	0.053	0.087	0.015
7p	0.536	0.189	0.464	0.527	0.028	0.042	0.042	0.018
8p	0.918	0.196	0.204	0.706	0.049	0.030	0.043	0.000
9p	0.491	0.270	0.545	0.167	0.041	0.040	0.061	0.018
10p	0.718	0.300	0.385	0.167	0.054	0.024	0.053	0.026
11p	0.495	0.301	0.592	0.176	0.049	0.049	0.069	0.039
12p	0.585	0.174	0.431	0.404	0.042	0.043	0.060	0.015
16p	0.802	0.118	0.459	0.076	0.178	0.097	0.195	0.012
17p	0.634	0.123	0.406	0.438	0.063	0.063	0.087	0.030
18p	0.500	0.500	0.500	0.333	0.023	0.017	0.023	0.000
19p	0.397	0.319	0.670	0.145	0.056	0.076	0.097	0.015
20p	0.597	0.173	0.542	0.264	0.077	0.078	0.106	0.000

Table 7: Ratios of selected tests for the breast cancer data set, per chromosome arm. Definitions used are in supplementary table 2.

arm	(CN sel & ME sel) /CN sel , colon	(CN sel & ME sel) /ME sel , colon	(CN sel & ME sel) /CN sel , breast	(CN sel & ME sel) /ME sel , breast
1q	0.059	0.114	0.163	0.203
2q	0.057	0.130	0.089	0.125
3q	0.052	0.141	0.075	0.095
4q	0.045	0.209	0.049	0.078
5q	0.045	0.177	0.125	0.640
6q	0.044	0.175	0.054	0.132
7q	0.115	0.397	0.072	0.121
8q	0.223	0.683	0.134	0.304
9q	0.047	0.183	0.049	0.057
10q	0.055	0.158	0.053	0.081
11q	0.065	0.138	0.191	0.210
12q	0.070	0.214	0.129	0.219
13q	0.238	0.764	0.161	0.375
14q	0.082	0.504	0.128	0.237
15q	0.058	0.280	0.077	0.151
16q	0.115	0.268	0.210	0.521
17q	0.056	0.104	0.197	0.259
18q	0.090	0.742	0.135	0.318
19q	0.097	0.088	0.115	0.096
20q	0.320	0.844	0.182	0.256
21q	0.056	0.180	0.067	0.061
22q	0.039	0.105	0.127	0.211
1p	0.075	0.270	0.150	0.197
2p	0.060	0.084	0.102	0.219
3p	0.091	0.282	0.114	0.221
4p	0.073	0.342	0.227	0.426
5p	0.094	0.296	0.053	0.043
6p	0.052	0.079	0.101	0.143
7p	0.078	0.356	0.027	0.018
8p	0.162	0.516	0.107	0.176
9p	0.021	0.094	0.081	0.083
10p	0.038	0.150	0.125	0.278
11p	0.063	0.123	0.178	0.176
12p	0.061	0.179	0.043	0.043
16p	0.140	0.355	0.300	0.550
17p	0.099	0.534	0.096	0.096
18p	0.113	0.857	0.000	0.000
19p	0.047	0.115	0.142	0.105
20p	0.210	0.745	0.192	0.189

Table 8: Proportions of overlapping selected tests, for both colon and breast cancer data sets. Definitions used are in supplementary table 2.

ProbeID	chr	Entrez	GSymbol	Start	colon CN p	colon ME p	colon joint p	breast CN p	breast ME p	breast joint p
A_23_P20503	8q	55630	*SLC39A4*	145608660	0.000	0.001	0.000	0.681	0.786	0.879
A_23_P36937	13q	26747	*NUFIP1*	44411834	0.000	0.000	0.000	0.220	0.037	0.074
A_23_P370030	20q	2686	*GGT7*	32912536	0.000	0.000	0.000	0.542	0.797	0.844
A_23_P40374	20q	128869	*PIGU*	32612437	0.000	0.000	0.000	0.319	0.276	0.393
A_23_P165962	20q	51098	*IFT52*	41658506	0.000	0.001	0.000	0.407	0.760	0.517
A_23_P204885	13q	64881	*PCDH20*	60882367	0.000	0.000	0.000	0.762	0.702	0.794
A_23_P218675	20q	10406	*WFDC2*	43542083	0.000	0.000	0.000	0.552	0.148	0.291
A_23_P17356	20q	78997	*GDAP1L1*	42341089	0.000	0.000	0.000	0.445	0.124	0.190
A_23_P142249	19q	11136	*SLC7A9*	38024941	0.750	0.001	0.001	0.364	0.223	0.278
A_23_P43490	9p	1029	*CDKN2A*	21958039	0.314	0.004	0.004	0.837	0.362	0.444

Table 9: Probes selected for illustration of gene-specific effects, with gene annotation and test results for both data sets.

2.4 Figures

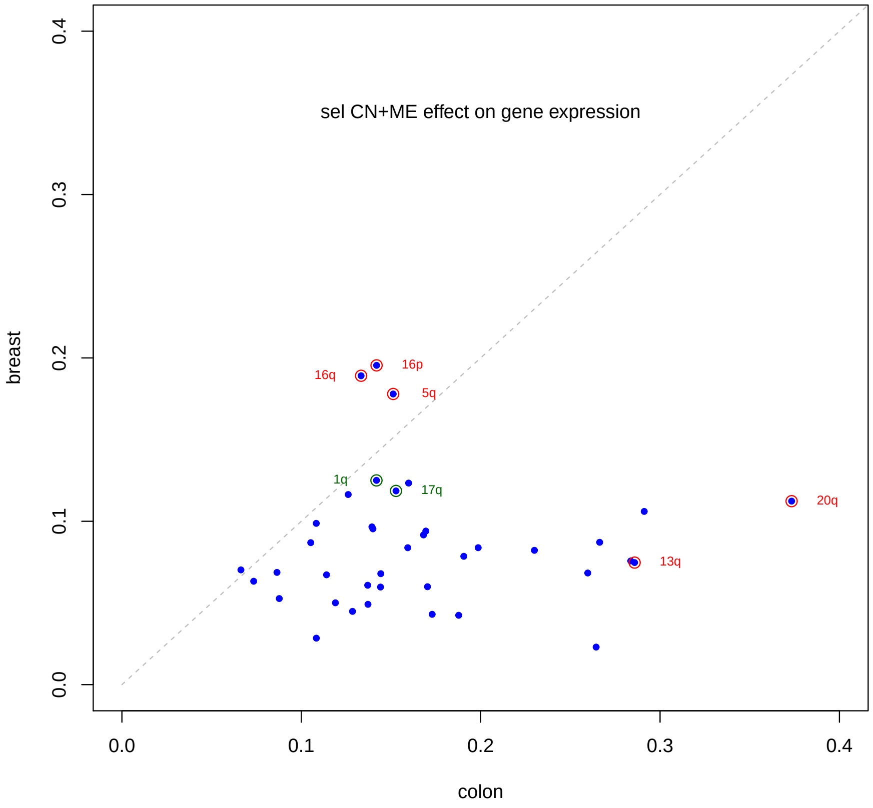

Figure 3: Proportions of selected associations between gene expression and both copy number and methylation, for colon and breast cancer

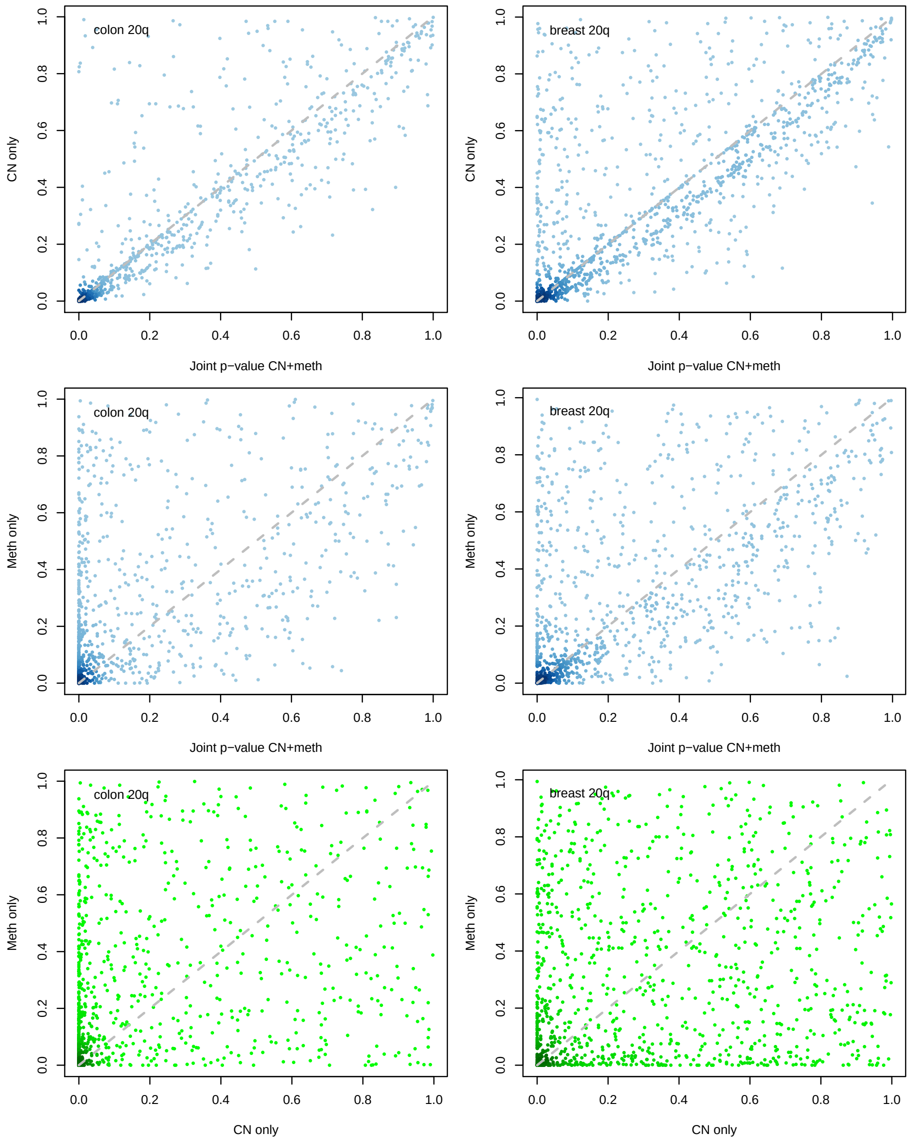

Figure 4: P-values obtained for the effects on 20q gene expression probes of: copy number only, methylation only and copy number and methylation jointly. Each graph is a scatterplot of joint p-values for copy number and methylation effects on gene expression (x-axis) and either copy number-effect p-values (y axis, top row), or methylation-effect p-values (y axis, middle row). The bottom row displays scatterplots of the individual test statistics. Left-hand side: colon cancer. Right-hand side: breast cancer. The point colour represents the density of points at that location in the scatterplot.

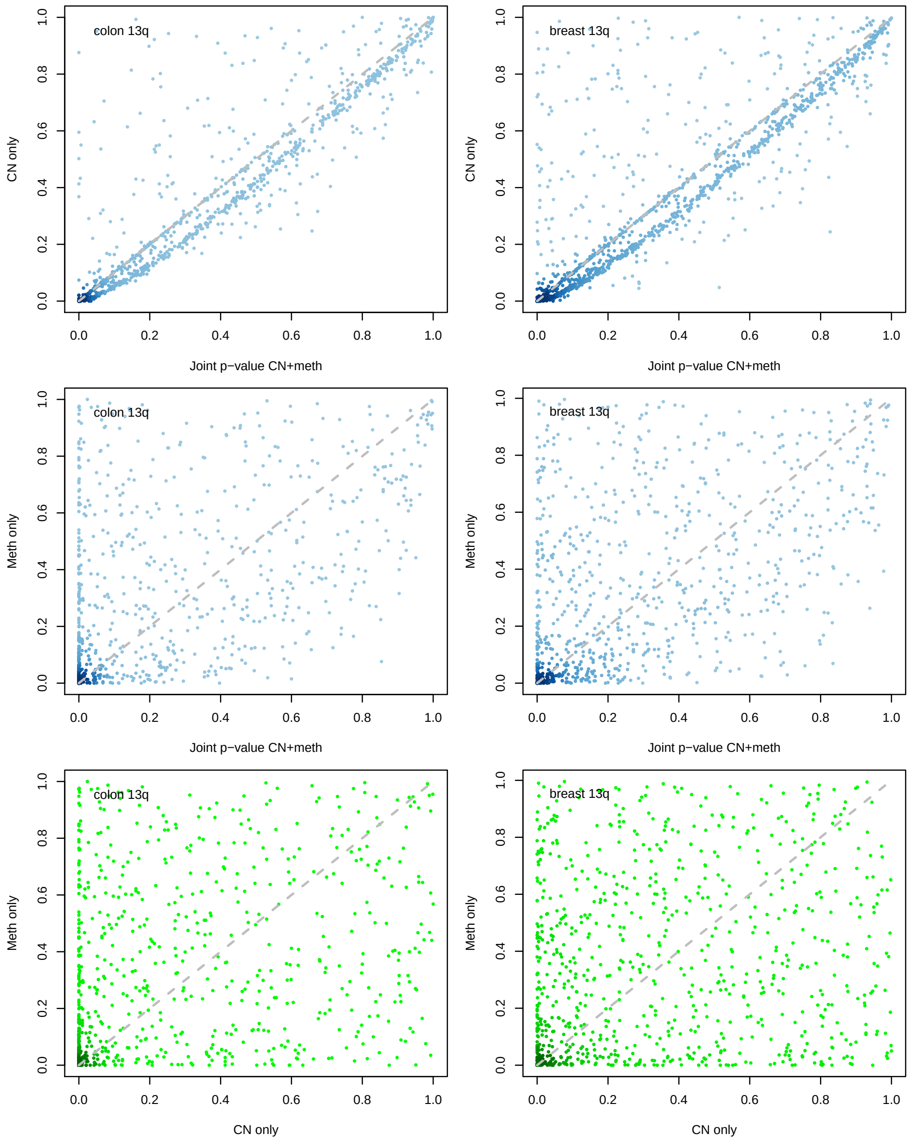

Figure 5: P-values obtained for the effects on 13q gene expression probes of: copy number only, methylation only and copy number and methylation jointly. Each graph is a scatterplot of joint p-values for copy number and methylation effects on gene expression (x-axis) and either copy number-effect p-values (y axis, top row), or methylation-effect p-values (y axis, middle row). The bottom row displays scatterplots of the individual test statistics. Left-hand side: colon cancer. Right-hand side: breast cancer. The point colour represents the density of points at that location in the scatterplot.

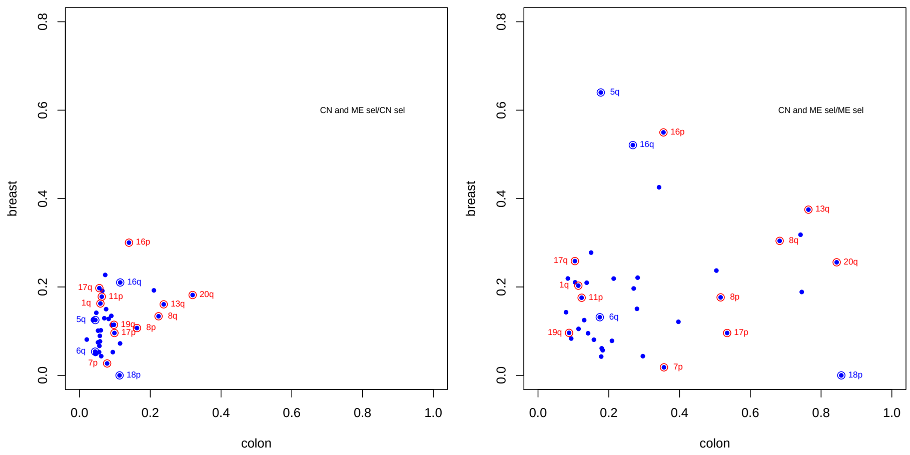

Figure 6: Proportions of overlap between selected tests with a single gene set. Left: proportions of mRNA probes with both CN test and ME test selected, relative to the total of selected CN tests. Right: proportions of mRNA probes both CN test and ME test selected, relative to the total of selected ME tests.

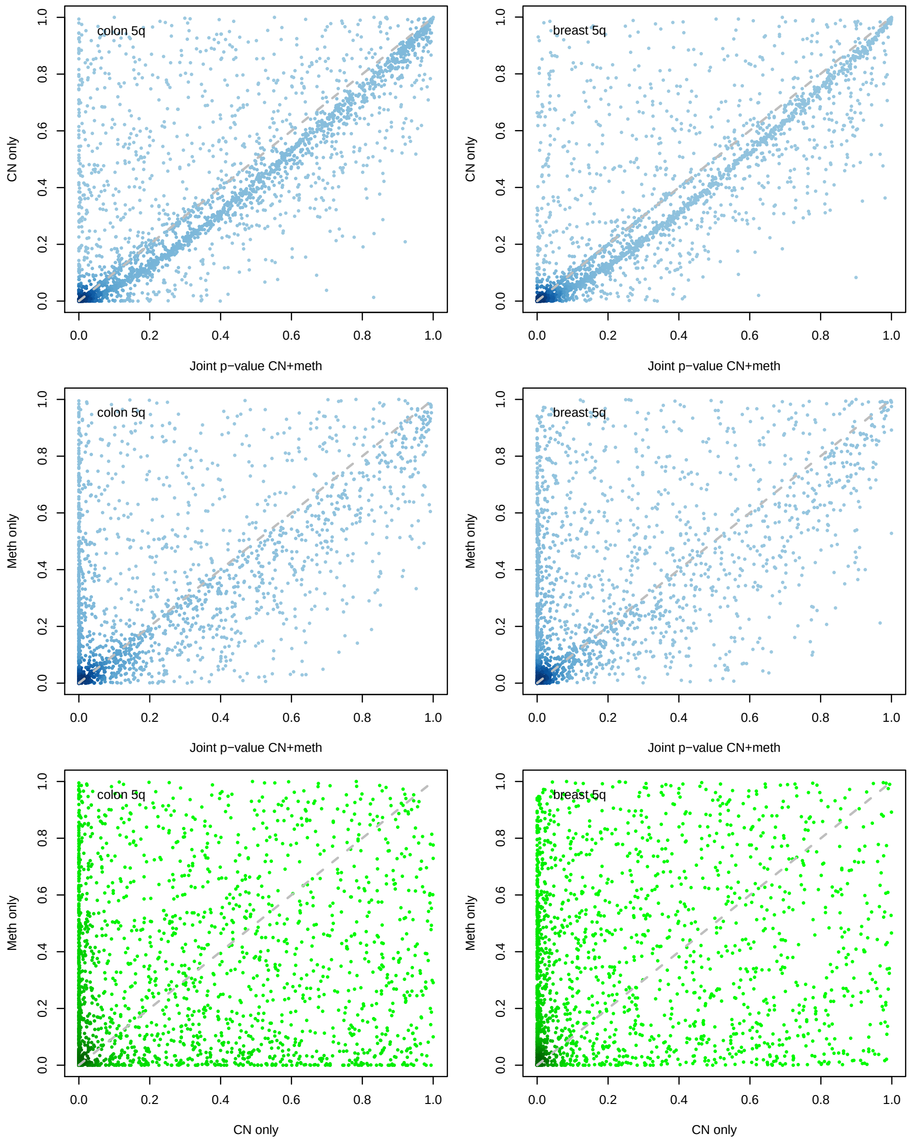

Figure 7: P-values obtained for the effects on 5q gene expression probes of: copy number only, methylation only and copy number and methylation jointly. Each graph is a scatterplot of joint p-values for copy number and methylation effects on gene expression (x-axis) and either copy number-effect p-values (y axis, top row), or methylation-effect p-values (y axis, middle row). The bottom row displays scatterplots of the individual test statistics. Left-hand side: colon cancer. Right-hand side: breast cancer. The point colour represents the density of points at that location in the scatterplot.

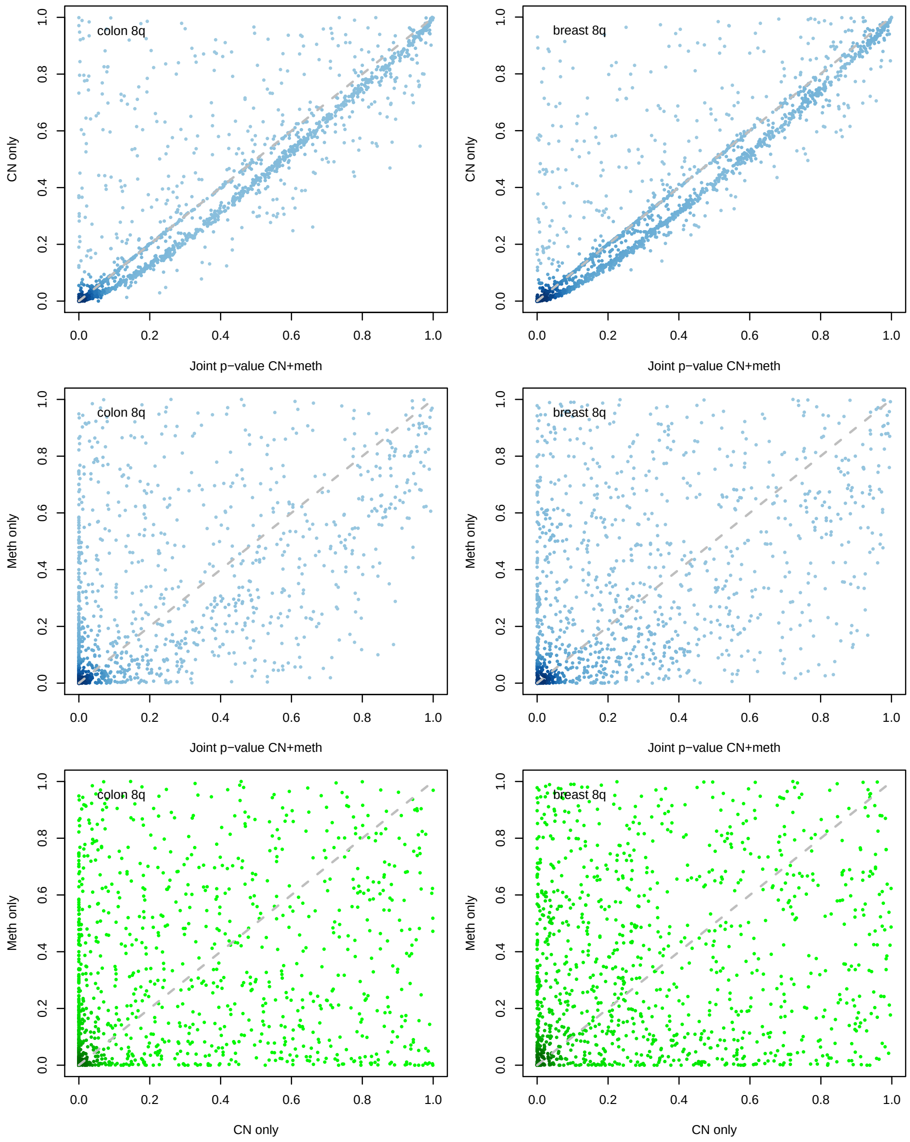

Figure 8: P-values obtained for the effects on 8q gene expression probes of: copy number only, methylation only and copy number and methylation jointly. Each graph is a scatterplot of joint p-values for copy number and methylation effects on gene expression (x-axis) and either copy number-effect p-values (y axis, top row), or methylation-effect p-values (y axis, middle row). The bottom row displays scatterplots of the individual test statistics. Left-hand side: colon cancer. Right-hand side: breast cancer. The point colour represents the density of points at that location in the scatterplot.

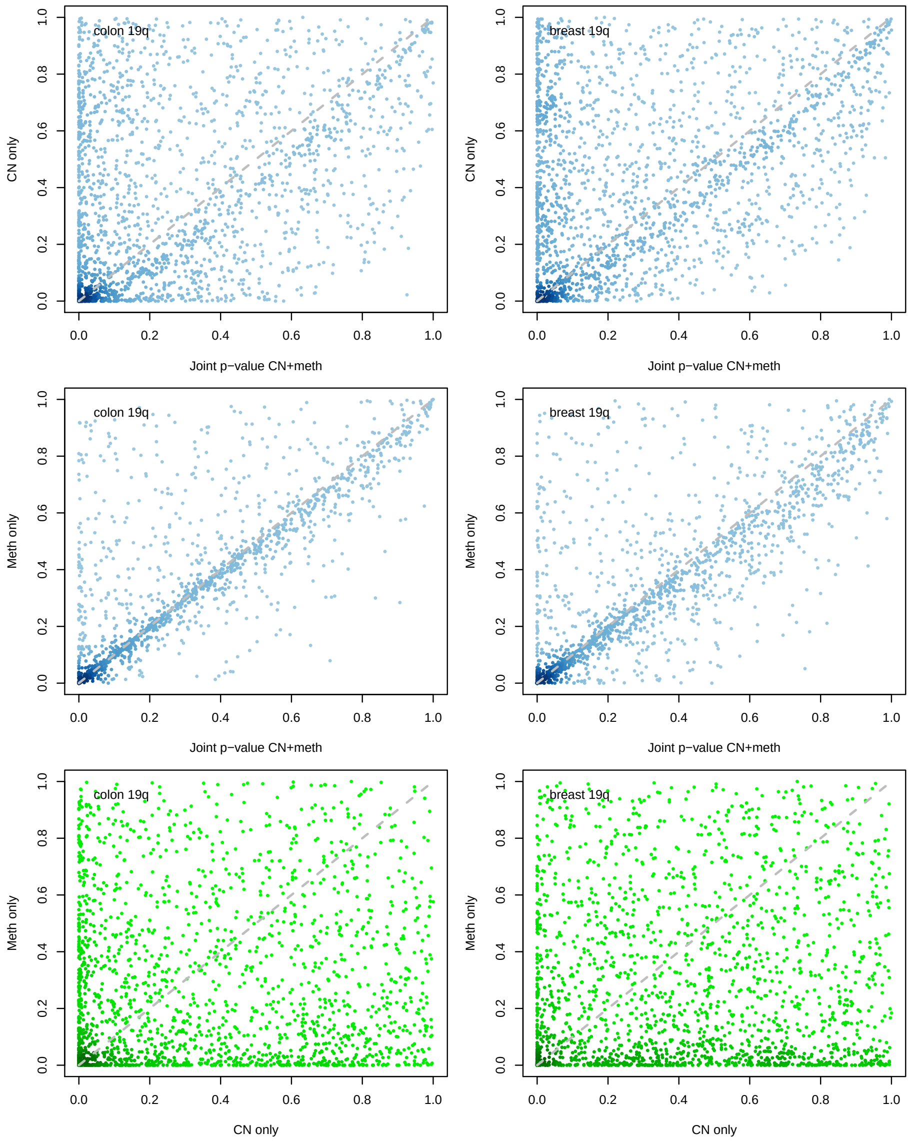

Figure 9: P-values obtained for the effects on 19q gene expression probes of: copy number only, methylation only and copy number and methylation jointly. Each graph is a scatterplot of joint p-values for copy number and methylation effects on gene expression (x-axis) and either copy number-effect p-values (y axis, top row), or methylation-effect p-values (y axis, middle row). The bottom row displays scatterplots of the individual test statistics. Left-hand side: colon cancer. Right-hand side: breast cancer. The point colour represents the density of points at that location in the scatterplot.

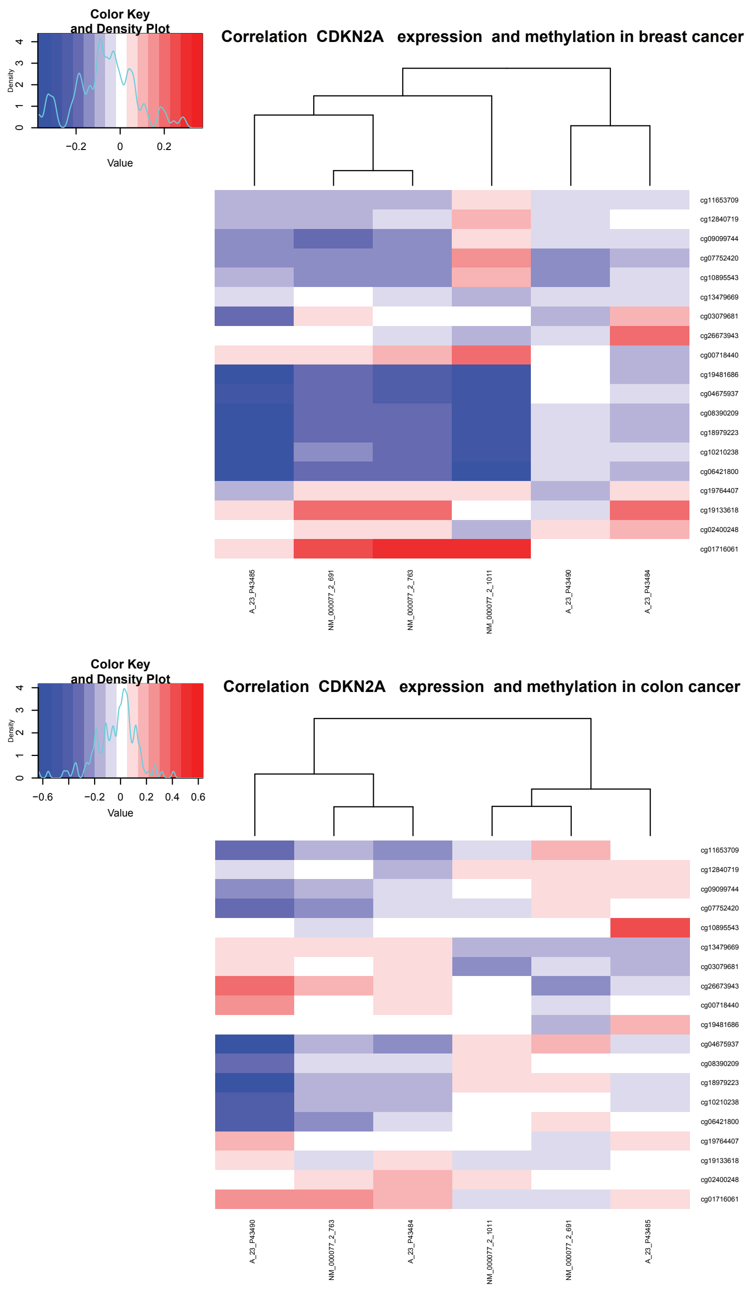

Figure 10: Heatmap of the Pearson correlation matrix between all 6 *CDKN2A* mRNA probes (columns) and the 19 methylation probes (rows) within 50Kb of the genes start site in either direction, for the colon (top) and breast (bottom) cancer data.

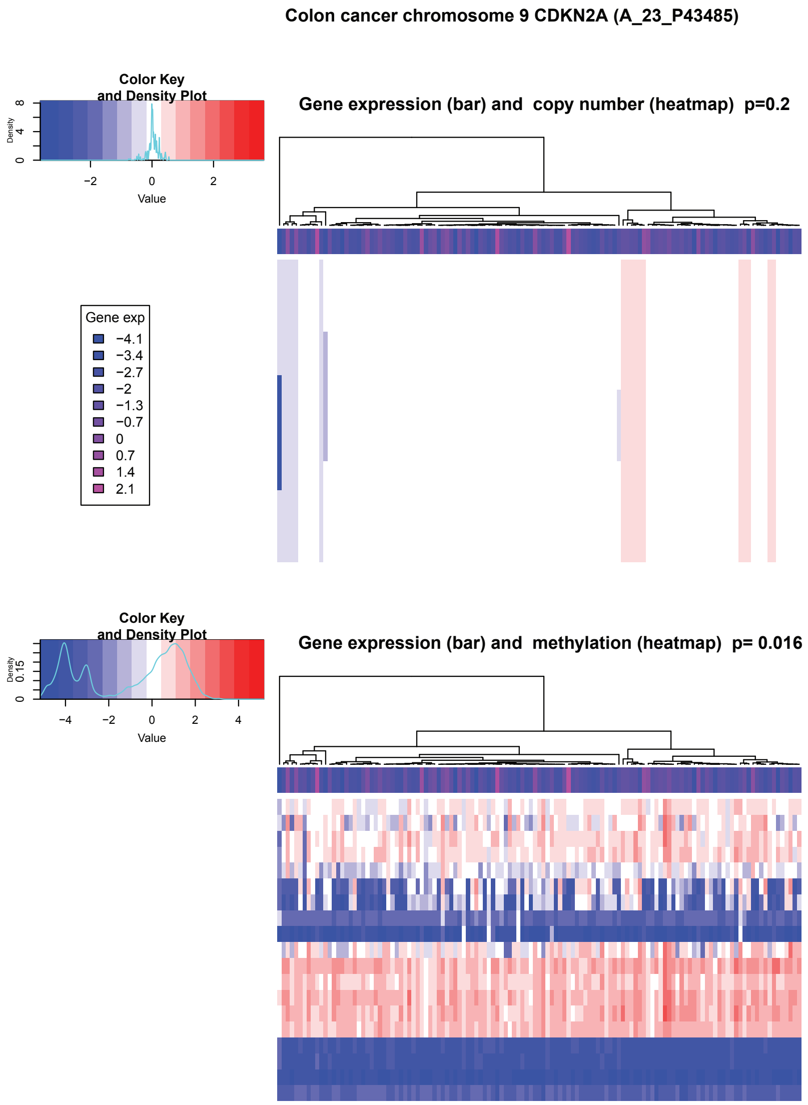

Figure 11: Colon cancer data heatmaps. Top: DNA copy number probes (rows), mapping to within 1Mb of *CDKN2A*'s start site in either direction, for 125 samples (columns). Bottom: methylation probes (rows), mapping to within 50Kb of *CDKN2A*'s start site in either, for 125 samples (columns). Samples are here in the same order as in the copy number heatmap (top). In both cases: a. probes are sorted according to their genome location, from top to bottom; b. the top-horizontal colour bar represents categorized mRNA expression values, corresponding to a single probe – see legend on top-left for a correspondence between values and colours.

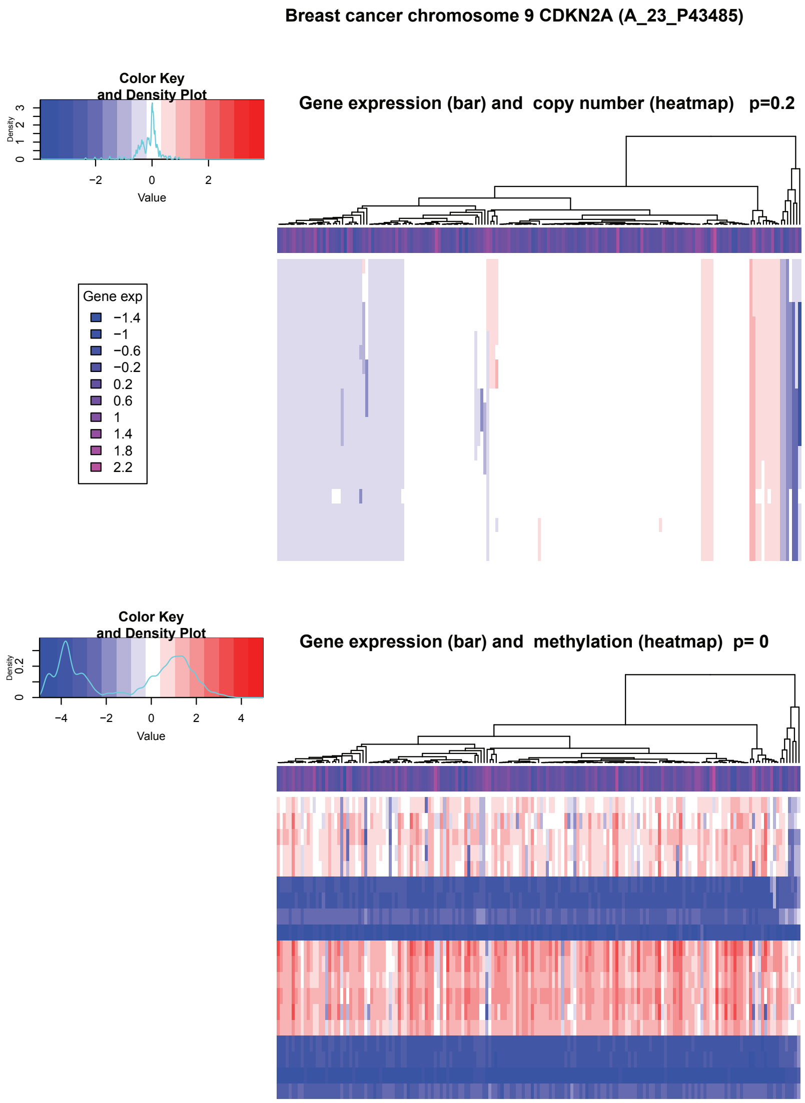

Figure 12: Breast cancer data heatmaps. Top: DNA copy number probes (rows), mapping to within 1Mb of *CDKN2A*'s start site in either direction, for 173 samples (columns). Bottom: methylation probes (rows), mapping to within 50Kb of *CDKN2A*'s start site in either, for 173 samples (columns). Samples are here in the same order as in the copy number heatmap (top). In both cases: a. probes are sorted according to their genome location, from top to bottom; b. the top-horizontal colour bar represents categorized mRNA expression values, corresponding to a single probe – see legend on top-left for a correspondence between values and colours.

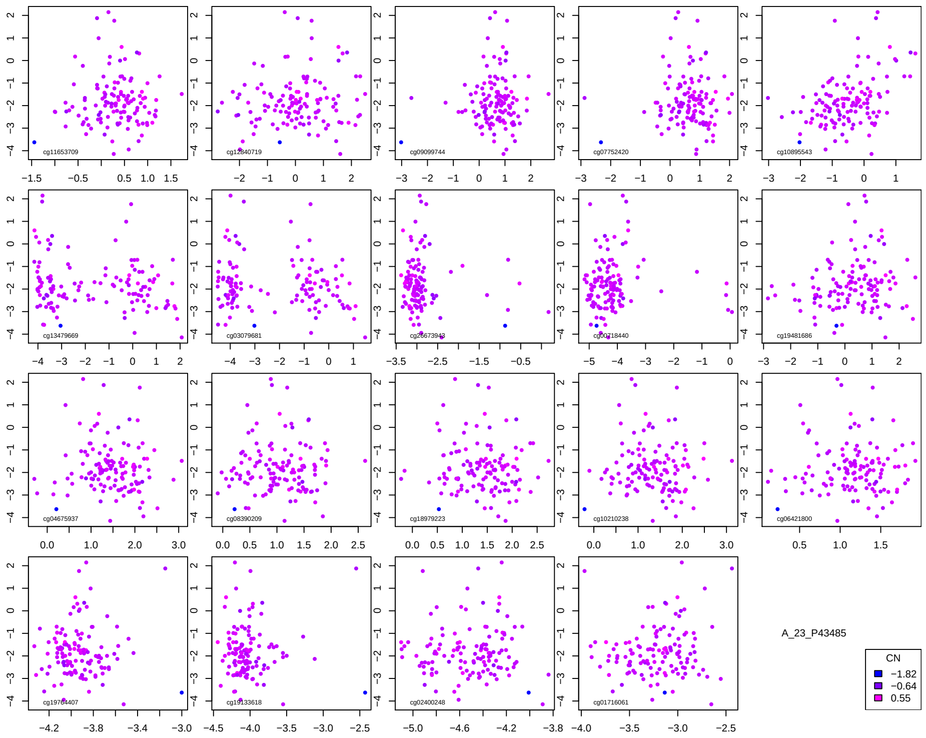

Figure 13: Scatterplots of each methylation probe in the covariate set for *CDKN2A* and one of its mRNA probes, for the colon cancer data set. The dot colour corresponds to the DNA copy number: as copy number values per sample are almost constant in the region, we summarize these by their median. See legend for the correspondence between colour and value ranges.

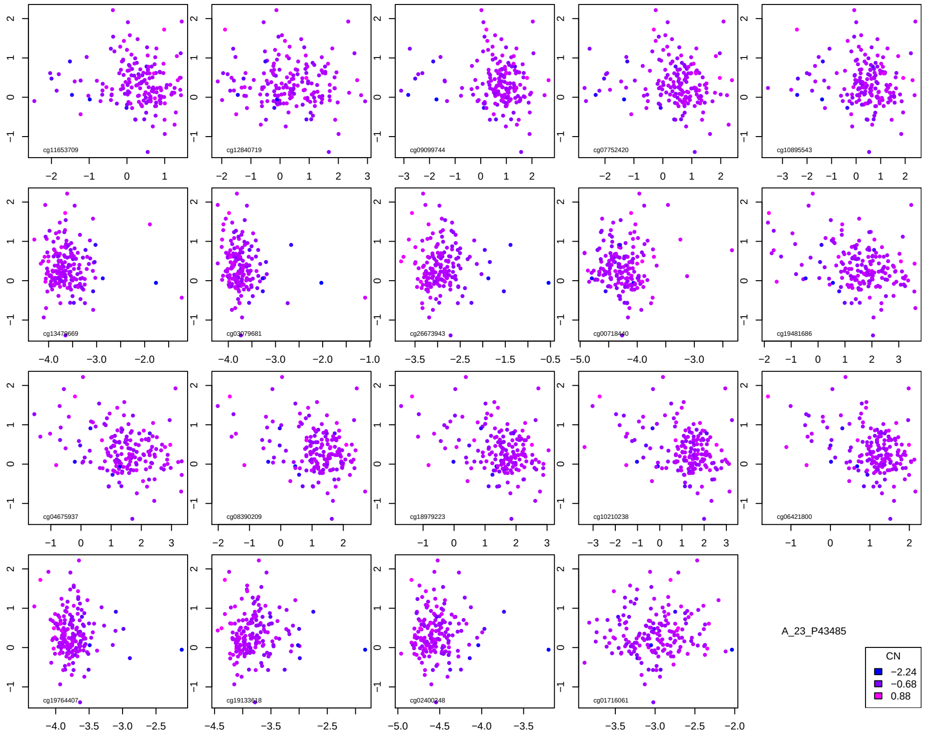

Figure 14: Scatterplots of each methylation probe in the covariate set for *CDKN2A* and one of its mRNA probes, for the breast cancer data set. The dot colour corresponds to the DNA copy number: as copy number values per sample are almost constant in the region, we summarize these by their median. See legend for the correspondence between colour and value ranges.

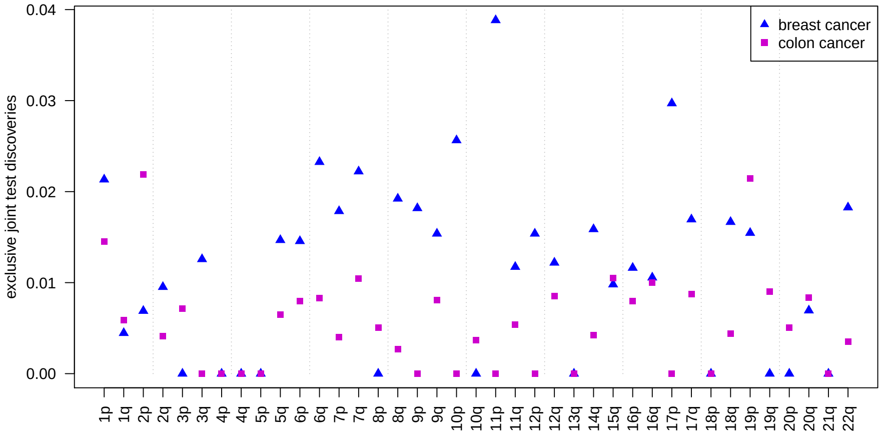

Figure 15: Proportion of all selected joint tests that are neither selected with CN test nor with ME test, per chromosome arm and cancer type.

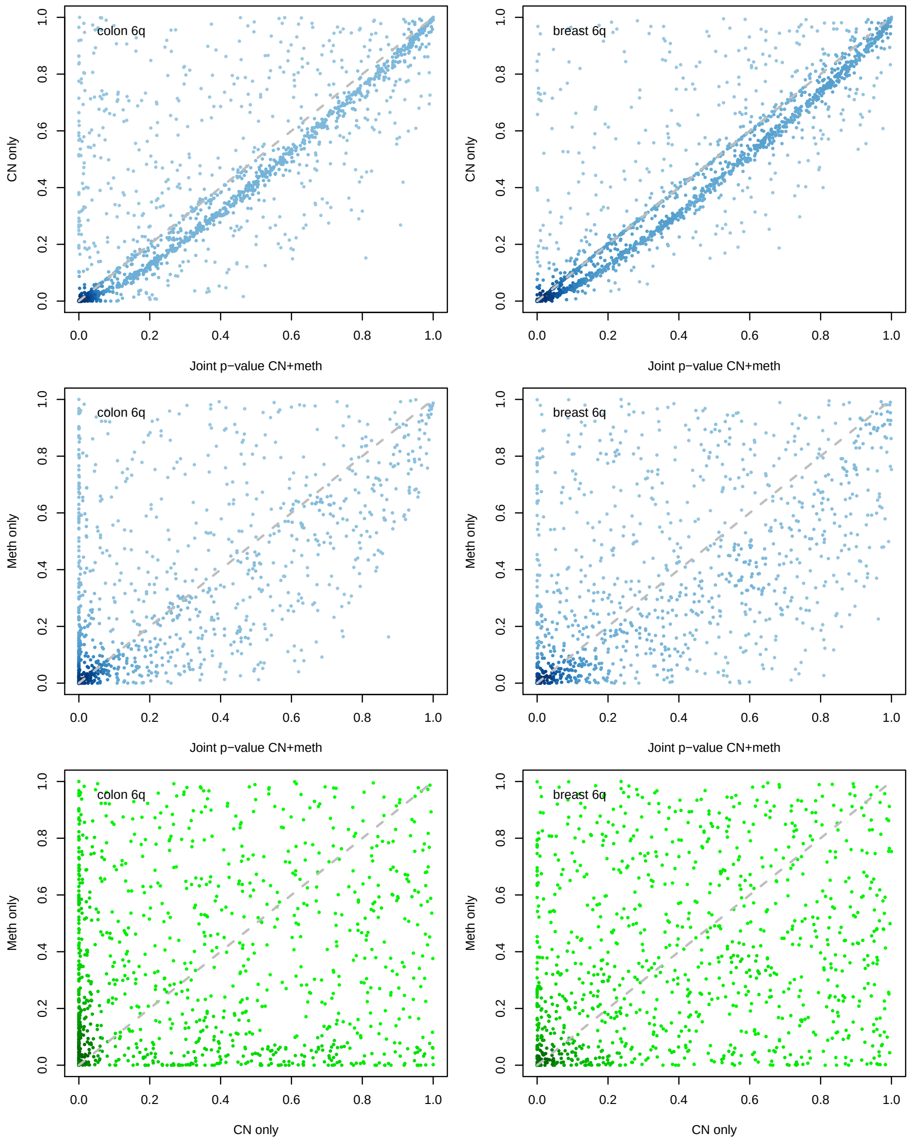

Figure 16: P-values obtained for the effects on 6q gene expression probes of: copy number only, methylation only and copy number and methylation jointly. Each graph is a scatterplot of joint p-values for copy number and methylation effects on gene expression (x-axis) and either copy number-effect p-values (y axis, top row), or methylation-effect p-values (y axis, middle row). The bottom row displays scatterplots of the individual test statistics. Left-hand side: colon cancer. Right-hand side: breast cancer. The point colour represents the density of points at that location in the scatterplot.

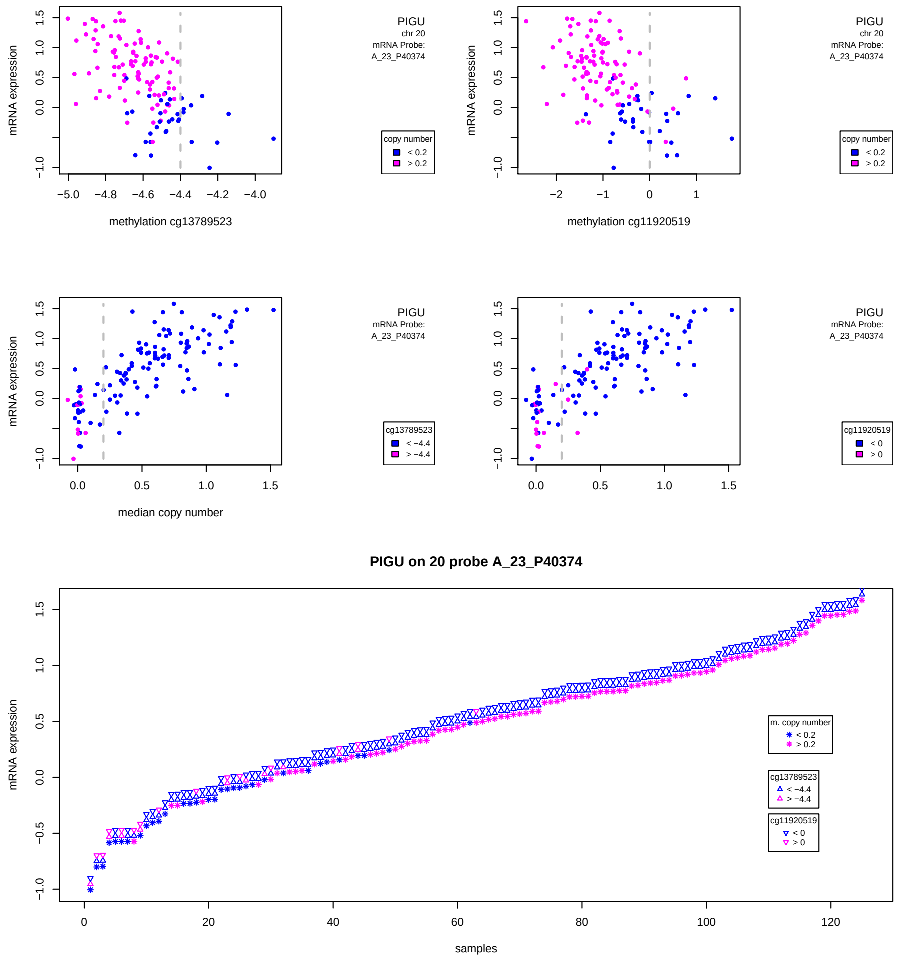

Figure 17: mRNA expression (y-axis) of one probe mapping to gene *PIGU* in the colon cancer data. Top graphs: methylation values (logit of beta values) for chosen probes within 50Kb of the gene start site; point colours represent the dychotomized copy number (see legend and dashed line in the graph right below), with blue corresponding to approximately diploid number of copies; the vertical dashed line represents the cut-off used to separate samples with more or less methylation. Middle graphs: median copy number values across all measurements within the 1 Mb region around the genes start site; point colours represent the dychotomized methylation (see legend and dashed line in the graph right above), with blue corresponding to less methylation; the vertical dashed line represents the cut-off used to separate samples approximately diploid from those. Bottom graph: mRNA expression for all samples, sorted from the smallest to the largest; three plotting symbols are used per sample to convey dychotomized copy number (star), methylation probe 1 (upwards triangle) and methylation probe 2 (downwards triangle); symbol colours are blue for lower values (diploid copy number, less methylation) or pink (copy gain, more methylation), as before.

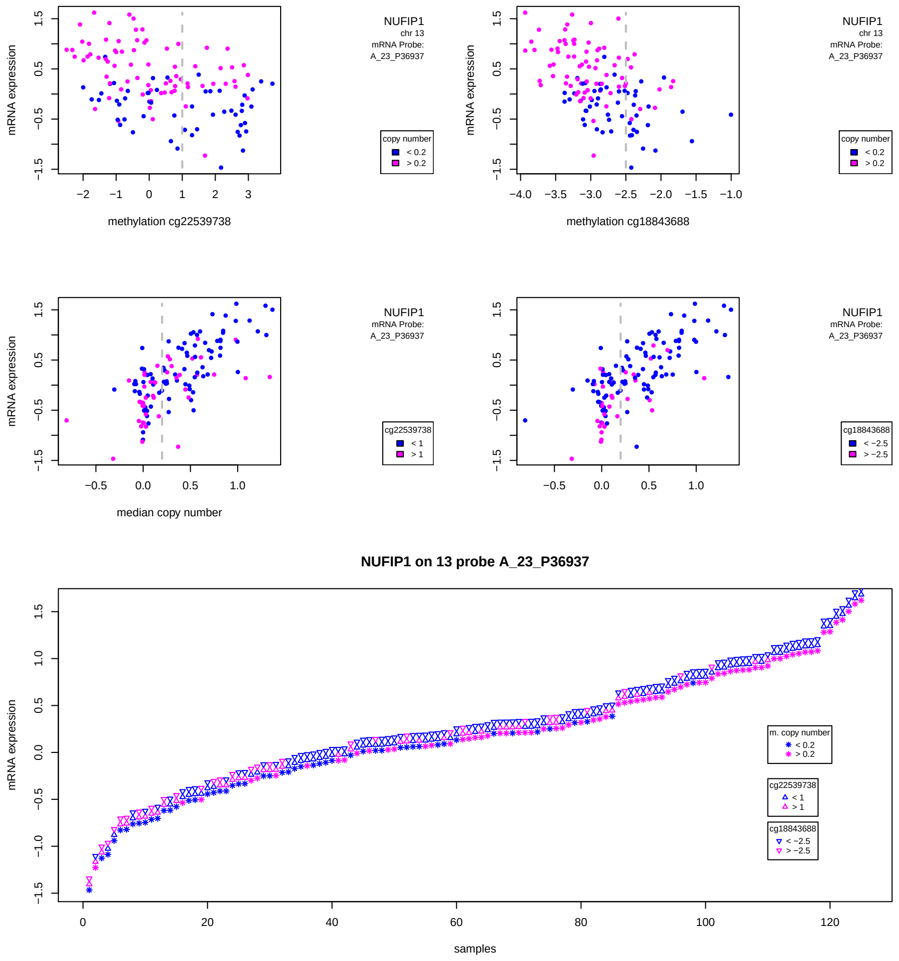

Figure 18: mRNA expression (y-axis) of one probe mapping to gene *NUFIP1* in the colon cancer data. Top graphs: methylation values (logit of beta values) for chosen probes within 50Kb of the gene start site; point colours represent the dychotomized copy number (see legend and dashed line in the graph right below), with blue corresponding to approximately diploid number of copies; the vertical dashed line represents the cut-off used to separate samples with more or less methylation. Middle graphs: median copy number values across all measurements within the 1 Mb region around the genes start site; point colours represent the dychotomized methylation (see legend and dashed line in the graph right above), with blue corresponding to less methylation; the vertical dashed line represents the cut-off used to separate samples approximately diploid from those. Bottom graph: mRNA expression for all samples, sorted from the smallest to the largest; three plotting symbols are used per sample to convey dychotomized copy number (star), methylation probe 1 (upwards triangle) and methylation probe 2 (downwards triangle); symbol colours are blue for lower values (diploid copy number, less methylation) or pink (copy gain, more methylation), as before.

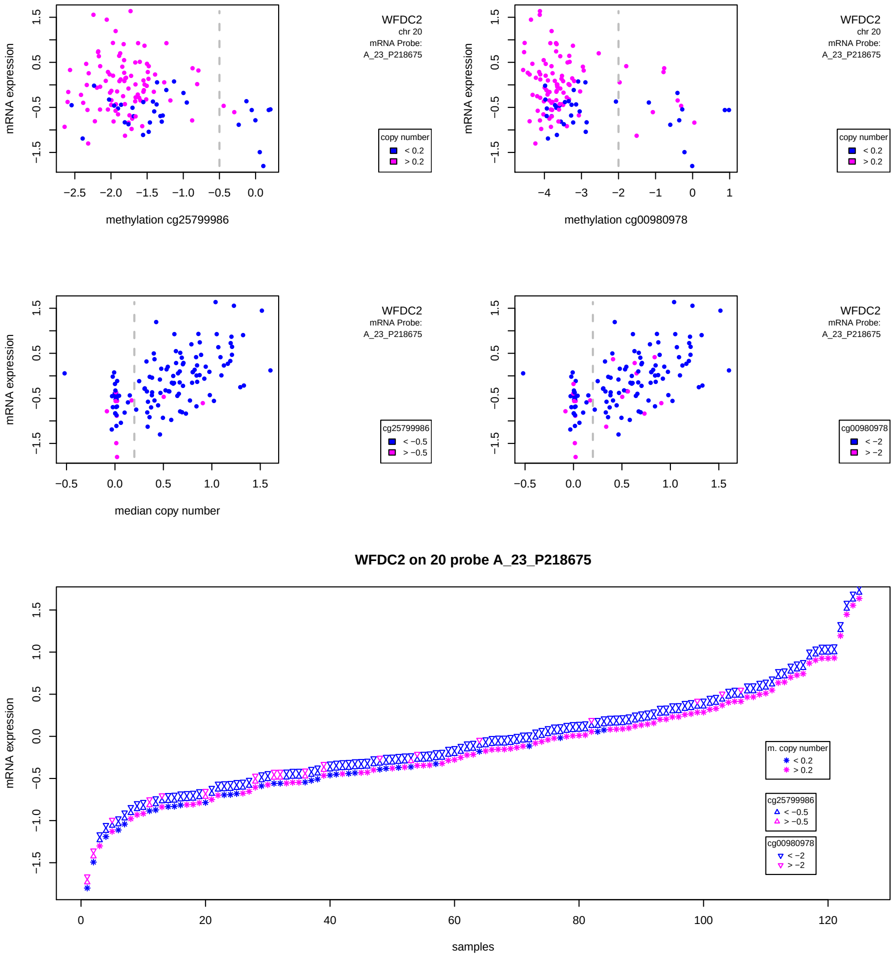

Figure 19: mRNA expression (y-axis) of one probe mapping to gene *WFDC2* in the colon cancer data. Top graphs: methylation values (logit of beta values) for chosen probes within 50Kb of the gene start site; point colours represent the dychotomized copy number (see legend and dashed line in the graph right below), with blue corresponding to approximately diploid number of copies; the vertical dashed line represents the cut-off used to separate samples with more or less methylation. Middle graphs: median copy number values across all measurements within the 1 Mb region around the genes start site; point colours represent the dychotomized methylation (see legend and dashed line in the graph right above), with blue corresponding to less methylation; the vertical dashed line represents the cut-off used to separate samples approximately diploid from those. Bottom graph: mRNA expression for all samples, sorted from the smallest to the largest; three plotting symbols are used per sample to convey dychotomized copy number (star), methylation probe 1 (upwards triangle) and methylation probe 2 (downwards triangle); symbol colours are blue for lower values (diploid copy number, less methylation) or pink (copy gain, more methylation), as before.

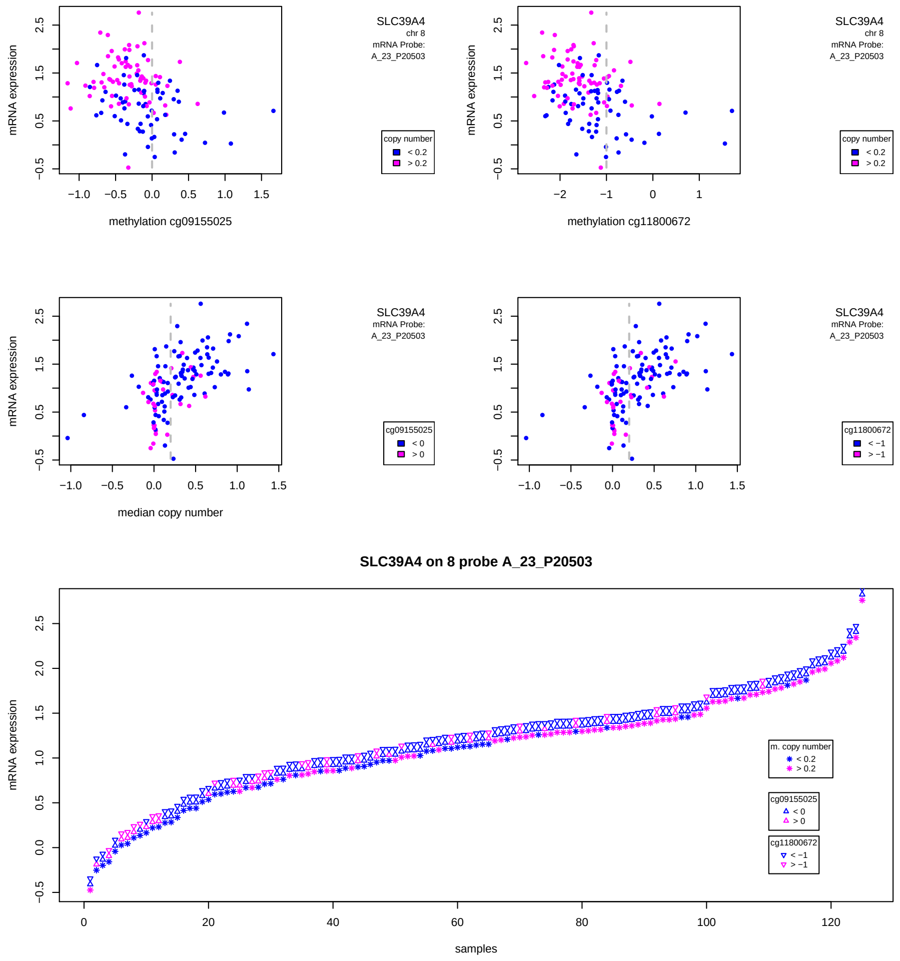

Figure 20: mRNA expression (y-axis) of one probe mapping to gene *SLC39A4* in the colon cancer data. Top graphs: methylation values (logit of beta values) for chosen probes within 50Kb of the gene start site; point colours represent the dychotomized copy number (see legend and dashed line in the graph right below), with blue corresponding to approximately diploid number of copies; the vertical dashed line represents the cut-off used to separate samples with more or less methylation. Middle graphs: median copy number values across all measurements within the 1 Mb region around the genes start site; point colours represent the dychotomized methylation (see legend and dashed line in the graph right above), with blue corresponding to less methylation; the vertical dashed line represents the cut-off used to separate samples approximately diploid from those. Bottom graph: mRNA expression for all samples, sorted from the smallest to the largest; three plotting symbols are used per sample to convey dychotomized copy number (star), methylation probe 1 (upwards triangle) and methylation probe 2 (downwards triangle); symbol colours are blue for lower values (diploid copy number, less methylation) or pink (copy gain, more methylation), as before.

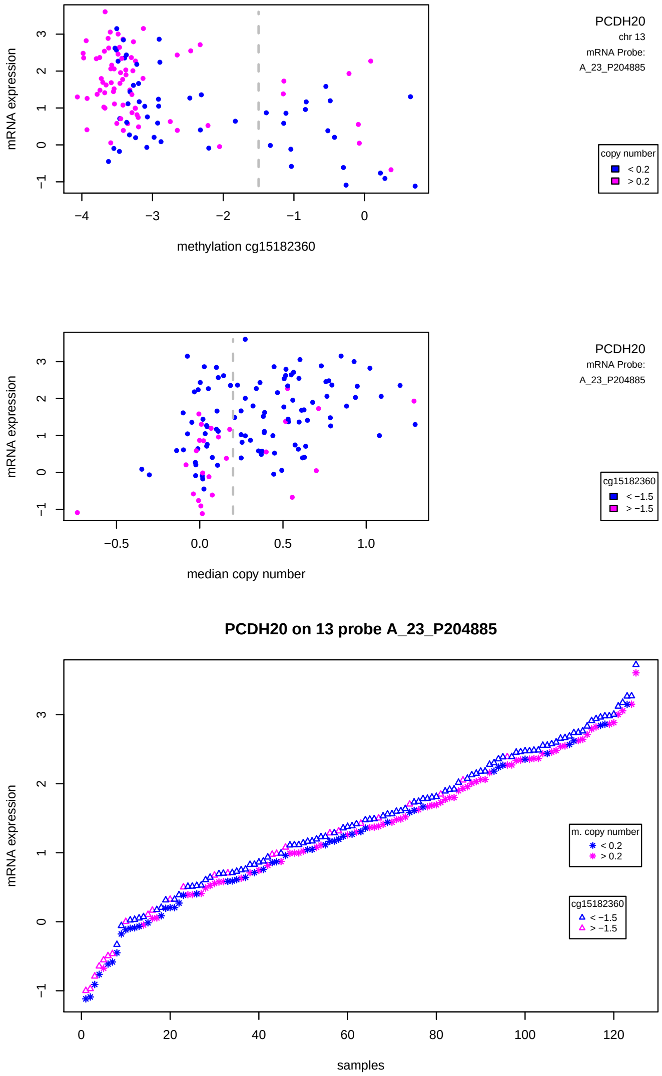

Figure 21: mRNA expression (y-axis) of one probe mapping to gene *PCDH20* in the colon cancer data. Top graphs: methylation values (logit of beta values) for chosen probes within 50Kb of the gene start site; point colours represent the dychotomized copy number (see legend and dashed line in the graph right below), with blue corresponding to approximately diploid number of copies; the vertical dashed line represents the cut-off used to separate samples with more or less methylation. Middle graphs: median copy number values across all measurements within the 1 Mb region around the genes start site; point colours represent the dychotomized methylation (see legend and dashed line in the graph right above), with blue corresponding to less methylation; the vertical dashed line represents the cut-off used to separate samples approximately diploid from those. Bottom graph: mRNA expression for all samples, sorted from the smallest to the largest; three plotting symbols are used per sample to convey dychotomized copy number (star), methylation probe 1 (upwards triangle) and methylation probe 2 (downwards triangle); symbol colours are blue for lower values (diploid copy number, less methylation) or pink (copy gain, more methylation), as before.

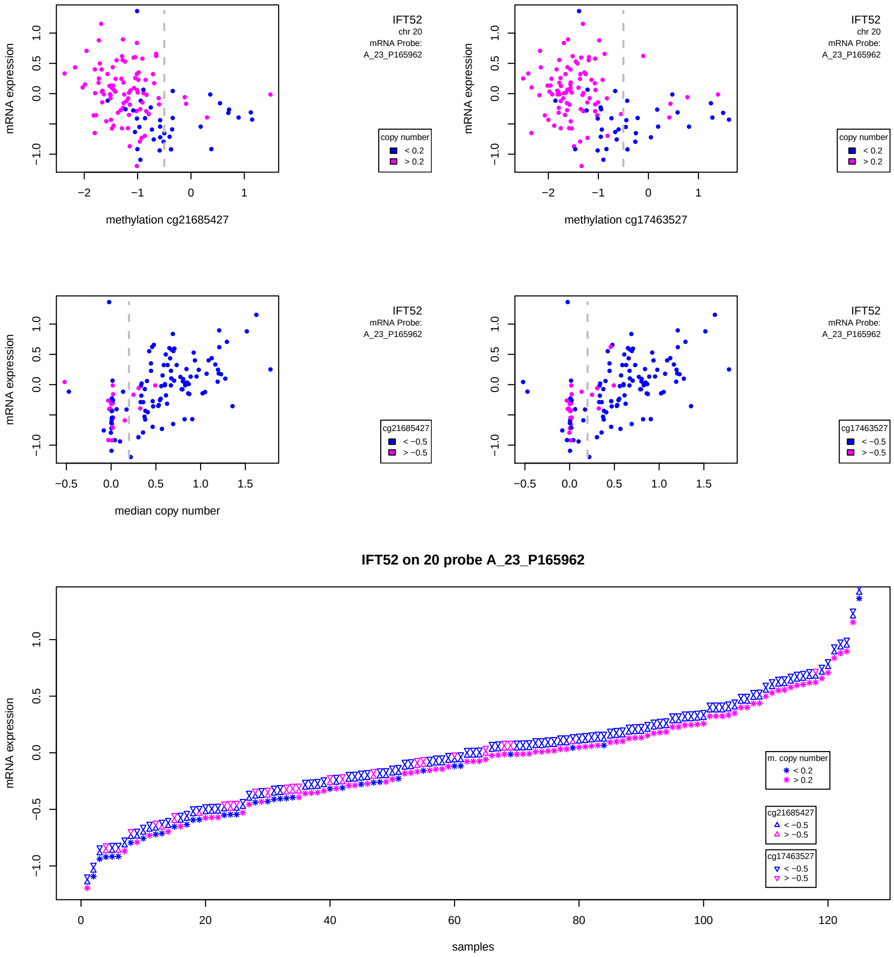

Figure 22: mRNA expression (y-axis) of one probe mapping to gene *IFT52* in the colon cancer data. Top graphs: methylation values (logit of beta values) for chosen probes within 50Kb of the gene start site; point colours represent the dychotomized copy number (see legend and dashed line in the graph right below), with blue corresponding to approximately diploid number of copies; the vertical dashed line represents the cut-off used to separate samples with more or less methylation. Middle graphs: median copy number values across all measurements within the 1 Mb region around the genes start site; point colours represent the dychotomized methylation (see legend and dashed line in the graph right above), with blue corresponding to less methylation; the vertical dashed line represents the cut-off used to separate samples approximately diploid from those. Bottom graph: mRNA expression for all samples, sorted from the smallest to the largest; three plotting symbols are used per sample to convey dychotomized copy number (star), methylation probe 1 (upwards triangle) and methylation probe 2 (downwards triangle); symbol colours are blue for lower values (diploid copy number, less methylation) or pink (copy gain, more methylation), as before.

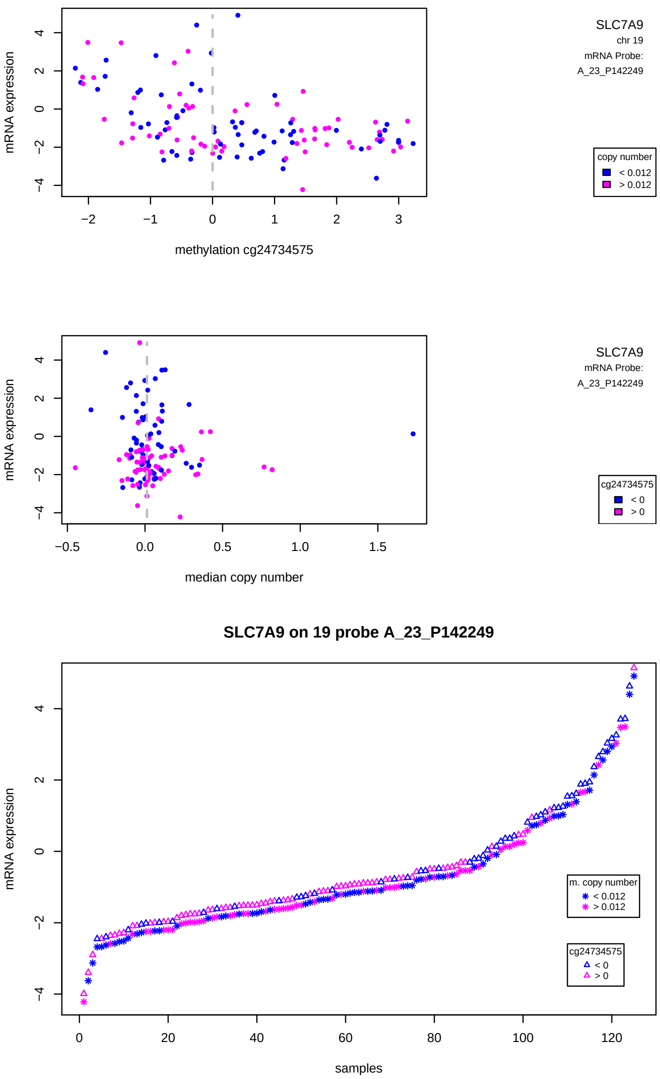

Figure 23: mRNA expression (y-axis) of one probe mapping to gene *SLC7A9* in the colon cancer data. Top graphs: methylation values (logit of beta values) for chosen probes within 50Kb of the gene start site; point colours represent the dychotomized copy number (see legend and dashed line in the graph right below), with blue corresponding to approximately diploid number of copies; the vertical dashed line represents the cut-off used to separate samples with more or less methylation. Middle graphs: median copy number values across all measurements within the 1 Mb region around the genes start site; point colours represent the dychotomized methylation (see legend and dashed line in the graph right above), with blue corresponding to less methylation; the vertical dashed line represents the cut-off used to separate samples approximately diploid from those. Bottom graph: mRNA expression for all samples, sorted from the smallest to the largest; three plotting symbols are used per sample to convey dychotomized copy number (star), methylation probe 1 (upwards triangle) and methylation probe 2 (downwards triangle); symbol colours are blue for lower values (diploid copy number, less methylation) or pink (copy gain, more methylation), as before.

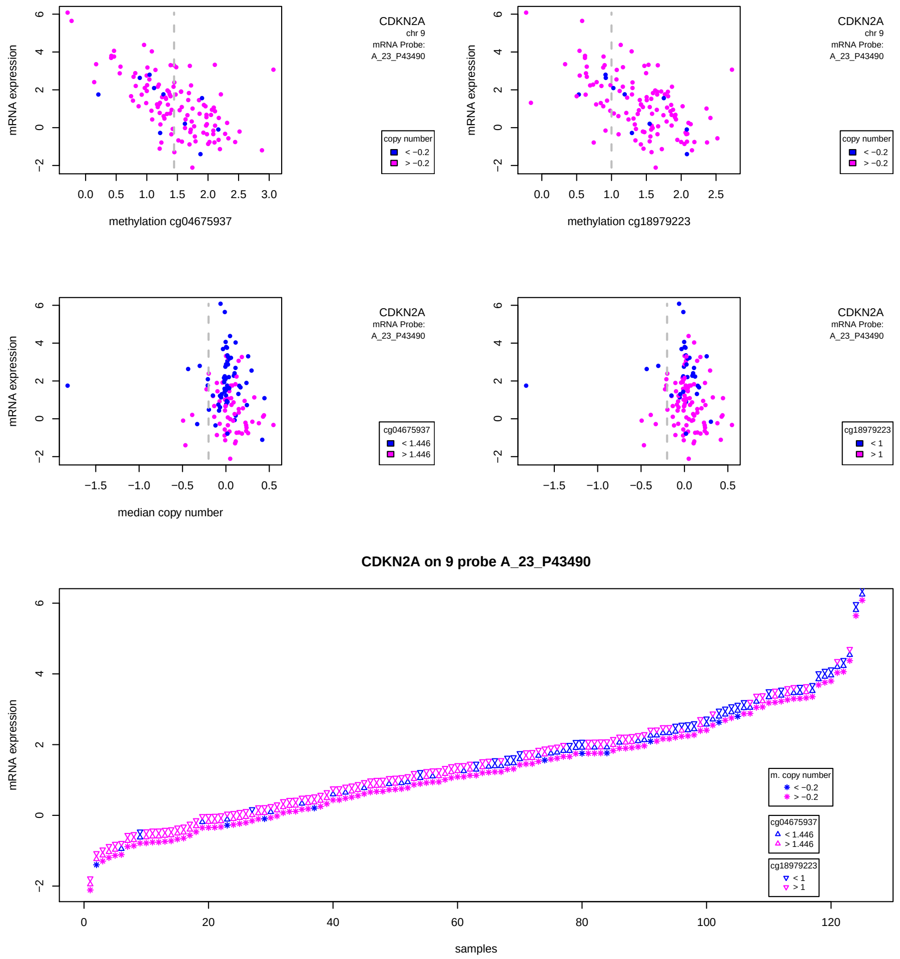

Figure 24: mRNA expression (y-axis) of one probe mapping to gene *CDKN2A* in the colon cancer data. Top graphs: methylation values (logit of beta values) for chosen probes within 50Kb of the gene start site; point colours represent the dychotomized copy number (see legend and dashed line in the graph right below), with blue corresponding to approximately diploid number of copies; the vertical dashed line represents the cut-off used to separate samples with more or less methylation. Middle graphs: median copy number values across all measurements within the 1 Mb region around the genes start site; point colours represent the dychotomized methylation (see legend and dashed line in the graph right above), with blue corresponding to less methylation; the vertical dashed line represents the cut-off used to separate samples approximately diploid from those. Bottom graph: mRNA expression for all samples, sorted from the smallest to the largest; three plotting symbols are used per sample to convey dychotomized copy number (star), methylation probe 1 (upwards triangle) and methylation probe 2 (downwards triangle); symbol colours are blue for lower values (diploid copy number, less methylation) or pink (copy gain, more methylation), as before.
